# Substrate Engineering of SWCNT *p*–*n* Junctions for Dual‐Mode Power Generation and Heat‐Flux Sensing

**DOI:** 10.1002/advs.76889

**Published:** 2026-07-30

**Authors:** Ryota Tamai, Hiroto Nakayama, Shuya Ochiai, Masayuki Takashiri

**Affiliations:** ^1^ Department of Materials Science Tokai University Hiratsuka Japan

**Keywords:** flexible thermoelectric generators, heat flux sensors, p–n junctions, photothermal conversion, single‐walled carbon nanotubes, substrate engineering

## Abstract

Autonomous thermoelectric power generation under uniform heating, without an external cold reservoir, remains an open challenge for self‐powered Internet‐of‐Things (IoT) sensors. This work shows that, for a fixed single‐walled carbon nanotube (SWCNT) *p–n* junction film, circuit, and adhesive‐bonded device structure, the substrate alone produces a substrate‐dependent transition between continuous power generation and high‐sensitivity heat‐flux sensing. Across three flexible substrates, cycloolefin polymer (COP), polyimide (PI), and polyethylene naphthalate (PEN), substrate infrared absorptivity and per‐unit‐area thermal inertia jointly govern the sign, magnitude, and temporal evolution of the output voltage. COP (absorbance 0.34; transmittance 45.8% at 8.8 µm) heats the film preferentially, sustaining a stable in‐plane gradient and delivering +0.38 mV at steady state. PI and PEN, with near‐complete absorption, transiently invert the gradient, producing excursions of −0.58 and −0.96 mV; the larger PEN response reflects 2.4‐fold greater thermal inertia, yielding −45 µV/K per junction pair. A transient thermal model and thermographic imaging reproduce these dynamics and indicate that the substrate optical contrast, rather than the interfacial factors common to all devices, principally governs the switching. For the present device structure and testing conditions, these results establish a substrate‐engineering framework for dual thermoelectric functions in a single SWCNT architecture.

## Graphical abstract

Through substrate engineering alone, a single‐walled carbon nanotube *p–n* junction device switches between two functions under uniform infrared irradiation. A low‐absorbance substrate sustains a central hot junction for continuous power generation, whereas high‐absorbance substrates transiently invert the temperature gradient for sensitive heat‐flux sensing—governed jointly by infrared absorptivity and thermal inertia.

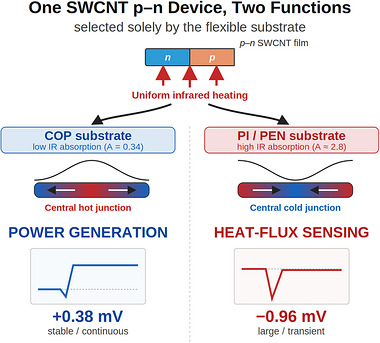

## 1 Introduction

The rapid expansion of Internet of Things (IoT) sensor networks across infrastructure, healthcare, and logistics [1, 2] is fundamentally constrained by the logistical and economic burden of battery replacement and physical wiring [3, 4, 5]. Among candidate autonomous power sources, thermoelectric conversion—the direct transformation of heat into electricity via the Seebeck effect—offers a continuous and reliable electrical supply from ubiquitous environmental heat [6, 7, 8, 9]. Recent efforts toward flexible and wearable thermoelectrics have expanded this concept across diverse material platforms, including emerging hydrogel‐based ionic thermoelectric systems for low‐grade heat harvesting [10].

Efficient power generation via the Seebeck effect, however, conventionally requires a sustained temperature difference between distinct hot and cold reservoirs [11, 12, 13], which is rarely available in ambient or industrial settings without bulky heat sinks or active cooling systems [14, 15, 16, 17, 18, 19]. Reported strategies to circumvent this constraint include radiative cooling into deep space [20, 21], the integration of phase‐change materials [22, 23], and our self‐sustaining single‐walled carbon nanotube (SWCNT) device that harnesses evaporative cooling while floating on water [24, 25]. More recently, we have been advancing a device architecture that autonomously establishes an internal temperature gradient—even under spatially uniform thermal irradiation—through deliberate engineering of thermal property mismatches between constituent materials [26, 27]. SWCNTs are particularly well‐suited to this concept: their exceptionally high infrared absorptivity enables efficient photothermal energy conversion [28, 29], so that asymmetric heating between the strongly absorbing film and its substrate can spontaneously produce an in‐plane temperature distribution. This raises the first core question addressed in this study: how can an internal in‐plane temperature gradient be generated within a thermoelectric device under spatially uniform infrared irradiation, in the absence of external hot and cold reservoirs?

A prerequisite for realizing such autonomous devices is the construction of robust, high‐performance *p*–*n* junctions [30]. Although SWCNTs intrinsically exhibit *n*‐type behavior immediately after synthesis [31, 32], they rapidly convert to *p*‐type upon atmospheric exposure through oxygen‐induced hole doping [33, 34, 35]. Our stabilization protocol utilizing the cationic surfactant dimethyldioctadecylammonium chloride (DODMAC) [36, 37, 38, 39] allows *n*‐type SWCNT films to maintain their thermoelectric characteristics for over two years [40], providing a reliable foundation for practical *p*–*n* junction devices.

Beyond material‐level stability, achieving true bifunctionality—where a single material platform can serve as either an autonomous power source or a high‐sensitivity sensor—requires a strategic shift from material‐level to substrate‐level engineering [41]. The “thermal property contrast”—specifically the mismatch in infrared absorptivity and thermal inertia between the SWCNT film and the supporting substrate—is hypothesized to be the primary factor governing device functionality [42, 43, 44]. Because the SWCNT film itself exhibits near‐complete infrared absorption (absorbance ∼2.97 at 8.8 µm) and thus responds uniformly to infrared irradiation regardless of substrate, it is the underlying substrate that must be engineered to modulate the internal heat flux [45], either maintaining a steady‐state temperature gradient for power generation or enabling a rapid transient response for sensing. Here, heat‐flux sensing capability denotes the generation of a large‐amplitude, rapid transient voltage signal in response to a sudden change in incident infrared irradiation, analogous to conventional thermopile‐based heat‐flux sensors [29]. This leads to the second core question: how do the infrared absorptivity and thermal inertia of the substrate determine not only the magnitude but also the direction of the internal temperature gradient, and hence the polarity of the thermoelectric output?

In this study, we address these two questions by systematically investigating the physical mechanisms through which substrate thermal properties govern device performance, thereby establishing design guidelines for substrate‐tailored bifunctional thermoelectrics. Using a series of commercially available flexible substrates with contrasting thermal properties—polyimide (PI), cycloolefin polymer (COP), and polyethylene naphthalate (PEN)—we correlate the spatial temperature distribution with the voltage output under uniform heating (first question) and isolate the respective roles of substrate infrared absorptivity and thermal inertia in determining the direction and magnitude of the internal temperature gradient (second question). Prior to evaluating device performance, we comprehensively characterized the active SWCNT films in terms of surface morphology, mechanical properties, and spatially resolved thermoelectric properties (Seebeck coefficient and electrical conductivity), establishing a material‐level foundation for interpreting the observed device behavior.

## 2 Experimental Details

**FIGURE 1:**
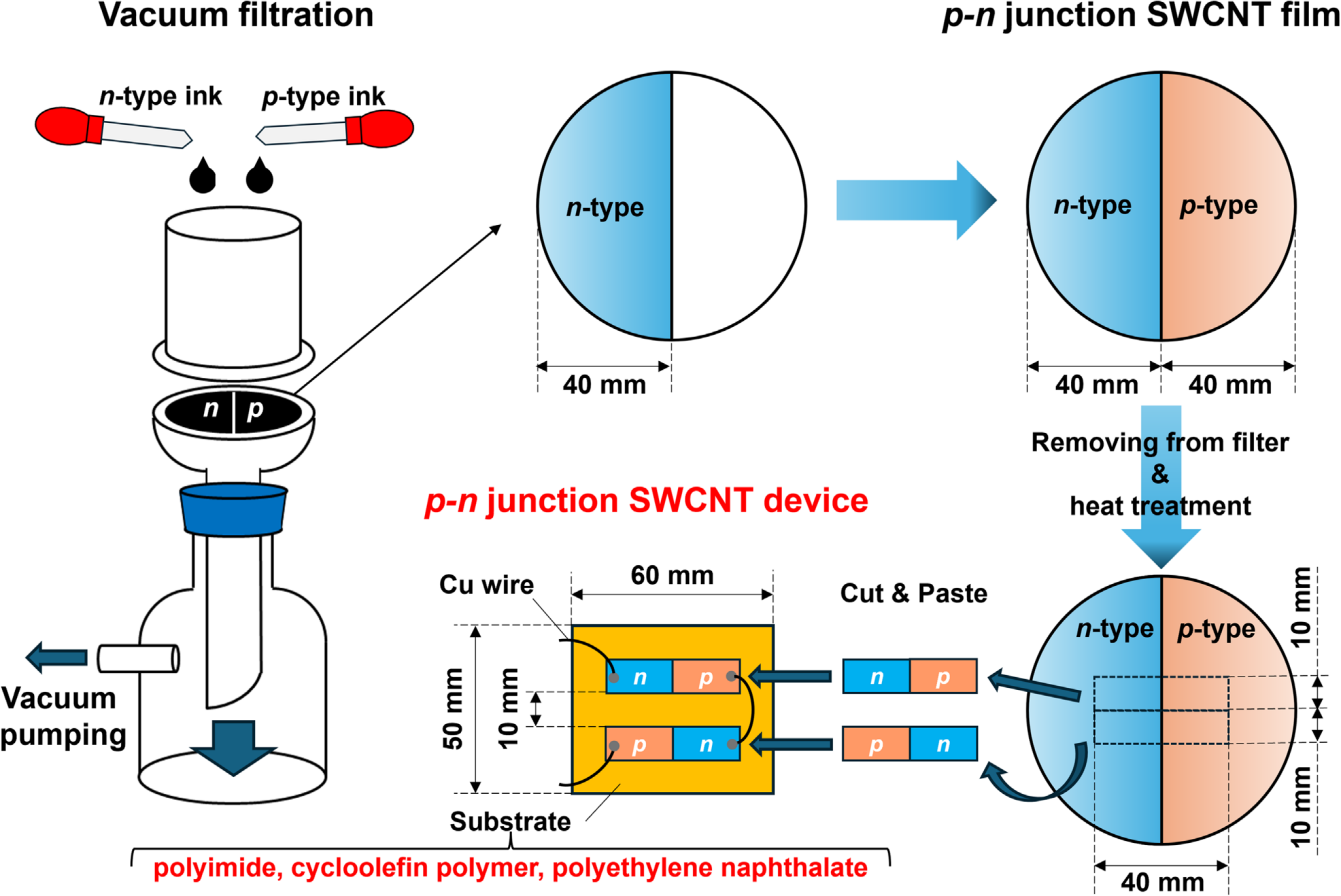
Schematic illustration of the fabrication process and device architecture of the *p–n* junction SWCNT thermoelectric device. Sequential vacuum filtration through a masked PTFE membrane (90 mm diameter) yields a monolithic circular film comprising an *n*‐type region (left half, 40 mm; DODMAC‐doped) and a *p*‐type region (right half, 40 mm; undoped). The composite film is then detached from the membrane, annealed under a reducing atmosphere (95% Ar / 5% H_2_, 473 K, 1 h), and diced into two rectangular strips (10 mm × 40 mm). The two strips are mounted on one of three flexible polymer substrates (60 mm × 50 mm)—polyimide (PI), cycloolefin polymer (COP), or polyethylene naphthalate (PEN)—with a 10 mm interval between them, in a series‐connected configuration in which the upper strip (*n*‐type left, *p*‐type right) is inverted relative to the lower strip (*p*‐type left, *n*‐type right). The *n*‐type terminal of the upper strip and the *p*‐type terminal of the lower strip are interconnected with copper wire and silver paste, and external copper leads are attached to the remaining terminals to complete the device.

Figure 1 illustrates the systematic fabrication process and the architecture of the *p–n* junction SWCNT devices. High‐purity single‐walled carbon nanotubes (SWCNTs; ZEONANO SG101, Zeon Corp.) served as the primary active material, dispersed in 99.5% ethanol. To induce *n*‐type characteristics, DODMAC (Tokyo Chemical Industry Co., Ltd.) was employed as the dopant. Three types of flexible substrates (60 mm × 50 mm) were evaluated: polyimide (PI; Kapton, DuPont; 125 µm thick), cycloolefin polymer (COP; ZEONOR, Zeon Corp.; 100 µm thick), and polyethylene naphthalate (PEN; Teonex, Teijin; 250 µm thick). The SWCNT films were prepared via vacuum filtration using a polytetrafluoroethylene (PTFE) membrane filter (90 mm diameter, 10 µm pore size; ADVANTEC).

To form the semiconducting regions, two distinct SWCNT dispersions were formulated. First, a common base dispersion was synthesized by mixing 80 mg of SWCNTs with 40 mL of ethanol, followed by probe sonication using an ultrasonic homogenizer (Branson Sonifier SFX 250) at 50% amplitude (nominal power of 200 W, ultrasonic frequency of 20 kHz, horn tip diameter of 12.7 mm) for 40 min in an ice bath to prevent overheating. This dispersion was subsequently divided into 8‐mL aliquots. To achieve *n*‐type characteristics, 1.25 wt.% DODMAC (relative to the SWCNT mass) was incorporated into the base dispersion, followed by an additional 10 min of sonication. For the *p*‐type regions, the base dispersion was used without further modification, exploiting the intrinsic *p*‐type behavior of SWCNTs resulting from atmospheric oxygen adsorption.

The integrated *p–n* junction films were produced via a sequential vacuum filtration method. To ensure distinct semiconducting domains without cross‐contamination, a precise masking protocol was implemented. Initially, the right half of the PTFE filter (from the right edge to the 40‐mm center line) was shielded, allowing the *n*‐type ink (with DODMAC) to be deposited onto the exposed left side via vacuum filtration. Subsequently, the mask was repositioned to cover the newly formed *n*‐type area (40 mm from the left edge), enabling the *p*‐type ink (without DODMAC) to be filtered onto the remaining right portion. The resulting composite film was detached from the membrane and subjected to thermal annealing at 473 K for 1 h under a reducing atmosphere (95% Ar and 5% H_2_) at atmospheric pressure.

Following heat treatment, two rectangular strips, each measuring 10 mm × 40 mm, were cut from the central area of the film. These strips were mounted onto one of the three selected substrates using Kapton double‐sided adhesive tape (No. 760H #25, Teraoka Seisakusho Co., 145 µm thick). As depicted in the device schematic of Figure 1, the two strips were aligned vertically with a 10‐mm interval between them. To facilitate a series electrical configuration, the upper strip was oriented with its *n*‐type region on the left, while the lower strip was inverted, placing its *p*‐type region on the left. The *n*‐type region of the upper strip and the *p*‐type region of the lower strip were then interconnected using copper wires and silver paste to form a series‐connected *p–n* junction. Finally, external copper leads were affixed to the remaining *n*‐ and *p*‐type terminals to enable precise voltage characterization.

The surface morphology and microstructure of the SWCNT films were investigated using scanning electron microscopy (SEM; S‐4800, Hitachi). Bundle‐diameter distributions were quantified from the SEM micrographs using image‐analysis software (ImageJ). For each region (*n*‐type, *p–n* junction interface, and *p*‐type), 100 bundles were randomly sampled across the field of view, and the diameter of each bundle was measured as its full width along a line drawn perpendicular to the local bundle axis at its midpoint, after spatial calibration against the embedded scale bar. The measured diameters were grouped into 25‐nm classes, and the mean and standard deviation (*n* = 100) were computed for each region; the resulting distributions are presented in Figure S1. To assess mechanical durability, tensile strength and breaking strain were measured at approximately 300 K using a precision testing machine (MX‐1000N‐FA, IMADA) [46, 47]. For these tests, five rectangular specimens (22 mm length × 2.5 mm width) were prepared from the 90‐mm‐diameter films to ensure statistical consistency and reproducibility. Optical properties were evaluated via Fourier‐transform infrared (FT‐IR) spectroscopy (IRSPIRIT‐T, Shimadzu), with absorbance recorded for both the SWCNT films and three substrates, as well as the Kapton double‐sided adhesive tape. The measurements were performed with a deliberately narrowed wavenumber interval in the vicinity of 8.8 µm (wavelength range 8.77–8.85 µm) in order to ensure the quantitative accuracy of the absorbance and transmittance values at this wavelength. The wavelength of 8.8 µm was selected because it is close to the peak of the blackbody radiation emitted by the hot‐plate at the operating temperature of 325 K, in accordance with Wien's displacement law (*λ*_max_ = 2898 µm·K/325 K ≈ 8.9 µm); the infrared radiation incident on the devices is most intense in this spectral region, so that the optical contrast among the substrates at this wavelength is the dominant factor governing the photothermal response. The thicknesses of the *p*‐type and *n*‐type SWCNT films were determined using a digital micrometer (DM0250, As‐One). The in‐plane thermal conductivity was determined from the thermal diffusivity, specific heat, and mass density. Thermal diffusivity was measured with an accuracy of ±5% using a non‐contact laser spot periodic heating radiative heat measurement system (TA33 Wave Analyzer, Bethe) [48]. The specific heat was determined by differential scanning calorimetry (DSC‐60PLUS, Shimadzu). The mass density of the SWCNT films was determined via the Archimedes method using an analytical balance (AP225WD, Shimadzu) with ethanol as the immersion medium. The thermal and optical properties of the SWCNT films and all three substrates, including thermal conductivity, infrared absorbance and transmittance at 8.8 µm, specific heat, and thickness, are summarized in Table 1. The original FT‐IR spectra from which the absorbance and transmittance values at 8.8 µm were directly extracted, including the spectrum of the Kapton double‐sided adhesive tape (Table S1), are presented in Figure S2.

**TABLE 1:** Thermal and optical properties of SWCNT films and flexible polymer substrates used in this study.

Materials	Thermal conductivity [W/(m·K)]	Absorbance (*λ* = 8.8 µm)	Transmittance (*λ* = 8.8 µm) [%]	Specific heat [J/(g·K)]	Thickness [µm]	Reference
*p*‐type SWCNT film	2.9	2.98	0.10	0.7	33	This work
*n*‐type SWCNT film	3.9	2.96	0.11	1.4	59	This work
Polyimide (PI)	0.12	2.84	0.15	1.1	125	Kapton (DuPont)
Cycloolefin polymer (COP)	0.1 – 0.2	0.34	45.8	1.2	100	ZEONOR (Zeon)
Polyethylene naphthalate (PEN)	0.15	2.71	0.20	1.3	250	Teonex (Teijin)

The in‐plane thermoelectric performance was characterized at nine distinct positions along the device's longitudinal axis, with a 5‐mm interval between measurements. The detailed protocols for these evaluations followed our established methodology reported previously [49, 50]. Briefly, the Seebeck coefficient was determined by applying a localized temperature gradient (Δ*T* = 1–4 K) using an attached heater. Two K‐type thermocouples were pressed onto the film surface with a 5‐mm separation to monitor Δ*T*, while the resulting thermoelectric voltage was recorded using a digital multimeter (R6561, ADVANTEST) and a temperature logging unit (GR‐3500, KEYENCE). The Seebeck coefficient was then calculated from the slope of the voltage–temperature linear relationship. Furthermore, the in‐plane electrical conductivity was measured at the corresponding locations using a four‐point probe system (RT‐70V, Napson).

To investigate power generation from *p*–*n* junction SWCNT devices under uniform heating conditions, the output voltage was monitored simultaneously with the temperature recorded using a thermocouple connected to a heat‐flow logger (LR8432, Hioki). The thermocouple was placed at the midpoint between the two *p*–*n* junction SWCNT films, corresponding to the geometric center of the device. The *p*–*n* junction SWCNT devices were mounted onto a hot‐plate stirrer (iSTIR HP320, NEUATION) and secured with Kapton tape along the edges. The output voltages of the SWCNT devices were acquired using a data logger (GL240, Graphtec). In addition, a thermographic camera (OPTXI40LTF20CFKT090, OPTRIS) was employed to visualize the overall temperature distribution and confirm the uniformity of heat flow across the device. The camera emissivity was set to *ε* = 0.95 for all measurements, consistent with the near‐blackbody infrared response of the SWCNT film surface, and this value was applied uniformly to the extraction of the regional temperatures in Figure 5. Thermographic images were acquired at three representative stages: (i) the heater‐off steady state immediately prior to activation, (ii) the transient period immediately following heater activation during the rapid temperature rise, and (iii) the heater‐on steady state after the device temperature had stabilized at 325 K. Voltage measurements were initiated, and 5 min after the start of data acquisition, the heater was turned on and set to a target temperature of 325 K. Voltage, temperature, and thermographic data were then continuously recorded for an additional 25 min following heater activation. For the reproducibility evaluation, three independently fabricated devices (*n* = 3) per substrate were instead recorded for 10 min after heater activation (to *t* = 15 min), a duration sufficient to capture the transient peak, the response time, and the subsequent plateau that define each operating mode; the representative device shown in Figure 4 was additionally monitored to *t* = 30 min to confirm that the steady‐state output remained stable.

## 3 Results

### 3.1 Characteristics of SWCNT Films

**FIGURE 2:**
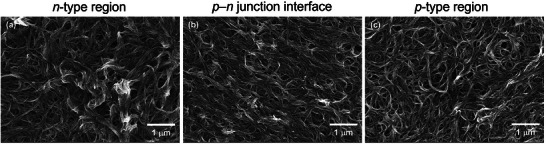
Scanning electron microscopy (SEM) images of the SWCNT film surface in the (a) *n*‐type region, (b) *p*–*n* junction interface, and (c) *p*‐type region. Scale bars: 1 µm. The *n*‐type region (a) exhibits a coarsened fibrous network with enlarged inter‐bundle voids and the largest mean bundle diameter (123 ± 85 nm, *n* = 100), attributable to encapsulation of individual bundles by DODMAC molecules, which promotes inter‐bundle aggregation. The junction interface (b) displays a noticeably denser morphology than the *n*‐type region, with a mean bundle diameter of 97 ± 68 nm; the partial filling of inter‐bundle spaces yields a compact, *p*‐type‐like packing, reflecting the graded blending of the two dispersions during sequential vacuum filtration. The *p*‐type region (c) shows a dense, homogeneous network with minimal inter‐bundle voids and the finest mean bundle diameter (93 ± 61 nm, *n* = 100), in marked contrast to the open architecture of the *n*‐type region. The full bundle‐diameter distributions (*n* = 100 per region) are presented in Figure S1.

Figure 2 presents the surface SEM images of the SWCNT films, illustrating the systematic morphological evolution across distinct regions. This structural transition arises from the physicochemical interactions between DODMAC—which functions simultaneously as a surfactant and an *n*‐type dopant—and the SWCNT surface, establishing a region‐dependent structural framework that underlies the electronic and thermal transport characteristics discussed in subsequent sections. In the *n*‐type region (Figure 2a), the effects of the surfactant and *n*‐type dopant on the network topology are most pronounced. The mean bundle diameter reaches 123 ± 85 nm (*n* = 100)—the coarsest among the three regions—accompanied by a marked enlargement of inter‐bundle voids. This morphological expansion is attributed to the encapsulation of individual SWCNT bundles by the additive molecules, which promotes inter‐bundle aggregation and yields a robust, highly interconnected fibrous architecture. The coarsened network morphology is anticipated to define the characteristic transport environment of the *n*‐type film, with implications for both carrier migration and thermal energy transfer addressed in the subsequent sections. At the *p*–*n* junction boundary region (Figure 2b), the network adopts an intermediate morphology, with a mean bundle diameter of 97 ± 68 nm—markedly finer than that of the *n*‐type region. In contrast to the open, void‐rich structure of the *n*‐type region, the boundary region exhibits a noticeably denser and more uniform packing, where partial occupation of inter‐bundle spaces by surfactant molecules yields a morphology approaching that of the *p*‐type region. The microscopic coexistence of *p*‐type and *n*‐type structural features defines a graded transition zone rather than a discrete boundary, a configuration that mitigates structural discontinuities and provides a physical basis for maintaining transport continuity across the heterojunction interface. In the *p*‐type region (Figure 2c), uniformly distributed bundles with a finer mean diameter of 93 ± 61 nm (*n* = 100) form a dense, homogeneous network with minimal inter‐bundle voids. The mean bundle diameters of the junction interface (97 ± 68 nm) and the *p*‐type region (93 ± 61 nm) are essentially indistinguishable, both being markedly finer than that of the *n*‐type region (123 ± 85 nm), consistent with the graded transition from the *n*‐type to the *p*‐type morphology across the junction. This high‐density configuration maximizes 3D network continuity and structural integrity, in marked contrast to the coarsened morphology of the *n*‐type region. The mechanical properties (tensile strength and breaking strain) of each region are summarized in Figure S3.

**FIGURE 3:**
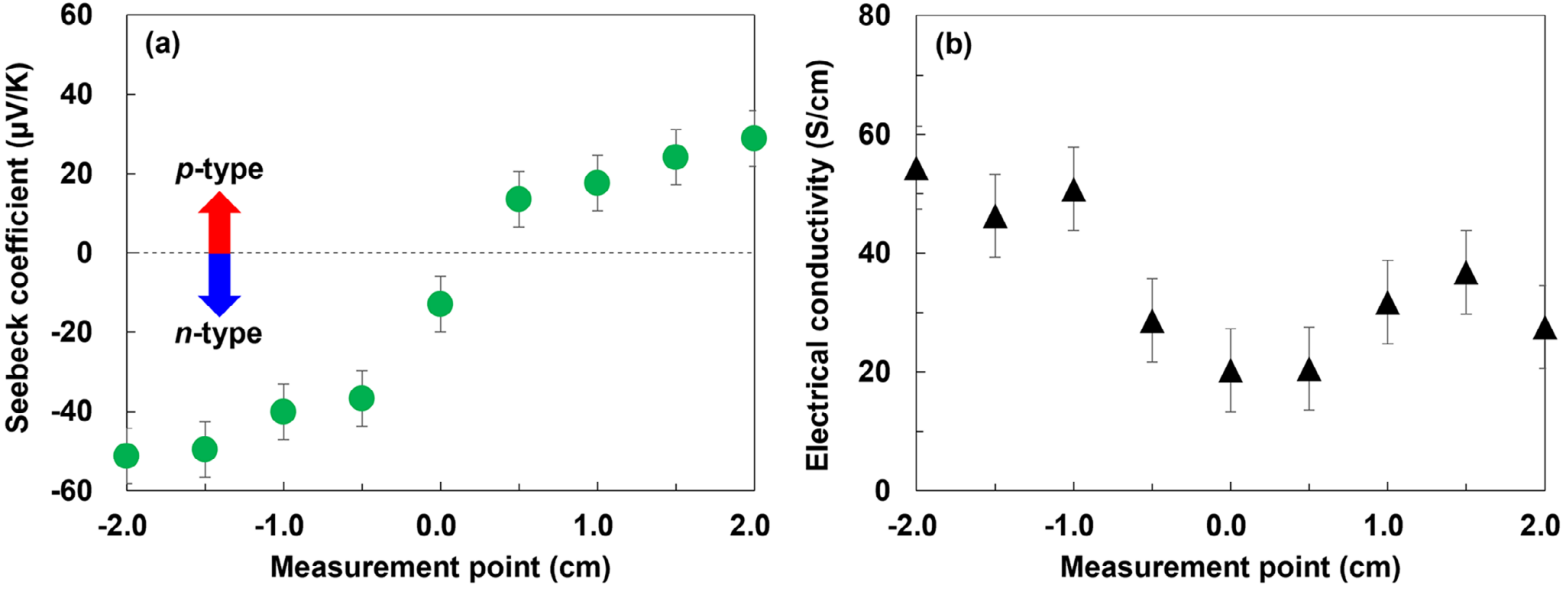
Spatial distribution of thermoelectric properties across the monolithic *p–n* junction SWCNT film measured at 0.5 cm intervals along the longitudinal axis (origin at the junction center). (a) Seebeck coefficient. The coefficient transitions smoothly from −52 µV/K in the *n*‐type region (−2.0 cm) through a steep polarity reversal near the junction interface to +29 µV/K in the *p*‐type region (+2.0 cm), indicating a continuous polarity gradient rather than an abrupt interface. The zero crossing occurs slightly positive of the geometric junction center, consistent with the graded interdiffusion zone formed during sequential vacuum filtration. The dashed horizontal line marks the zero baseline. (b) Electrical conductivity. Values reach 55 S/cm in the *n*‐type region and decrease to a minimum of 20 S/cm in the vicinity of the junction center (0 to +0.5 cm), reflecting the structural heterogeneity of the graded transition zone. Conductivity partially recovers to 27–37 S/cm in the *p*‐type region, remaining intermediate between the *n*‐type maximum and the junction minimum.

To characterize the spatial distribution of thermoelectric properties across the monolithic *p–n* junction film, the Seebeck coefficient and electrical conductivity were mapped as a function of measurement position, as presented in Figure 3. These transport characteristics are interpreted in light of the region‐dependent network morphologies established in Figure 2. Figure 3a presents the spatial profile of the Seebeck coefficient across the SWCNT film. In the *n*‐type region (−2.0 to −0.5 cm), the coefficient is consistently negative, with a value of −52 µV/K at −2.0 cm, reflecting well‐established electron‐dominated transport. A gradual increase toward zero is observed across this region, with a calculated gradient of 9.7 µV/(K·cm), consistent with the progressive attenuation of *n*‐type doping away from the film center. A pronounced transition occurs between −0.5 and +0.5 cm, where the Seebeck coefficient shifts steeply at a calculated rate of 50.2 µV/(K·cm), passing through approximately −13 µV/K at the 0 cm position before crossing into positive territory. This steep polarity reversal demarcates the *p*–*n* junction interface and is attributed to the interfacial blending of the two dispersions during vacuum filtration, which gives rise to the graded transition zone identified morphologically in Figure 2b. In the *p*‐type region (0.5 to 2.0 cm), the coefficient continues to increase at a calculated gradient of 10.2 µV/(K·cm), reaching +29 µV/K at 2.0 cm, indicative of stable hole‐dominated transport consistent with the dense, homogeneous network morphology of this region (Figure 2c). Collectively, these results indicate the formation of a continuous *p*–*n* junction characterized by a smooth polarity gradient rather than an abrupt interface. The batch‐to‐batch reproducibility of this graded interface was verified by fabricating two additional monolithic *p–n* junction films in independent batches under identical sequential vacuum filtration conditions. Both films reproduced the characteristic spatial profile of the Seebeck coefficient, varying monotonically from −55 µV/K in the *n*‐type region to +30 to +45 µV/K in the *p*‐type region, with the polarity crossover located within ±0.5 cm of the geometric junction position and the steep transition confined to the −0.5 to +0.5 cm region; all three batches followed the same monotonic n‐to‐p profile, exhibiting consistent polarity and comparable magnitudes at every measurement position (Figure S4). These results indicate that the graded interface is a reproducible consequence of the sequential vacuum filtration process, governed self‐consistently by the masking geometry rather than by incidental variations of an individual specimen. With respect to environmental stability, the DODMAC‐stabilized *n*‐type region has been reported to retain its thermoelectric performance for over two years [40], consistent with the robust air stability documented in our previous work and attributable to the encapsulation‐induced structural robustness of the *n*‐type network discussed in Figure 2a.

Figure 3b presents the spatial distribution of electrical conductivity across the SWCNT film, with values ranging from 20 to 55 S/cm. The *n*‐type region (−2.0 to −0.5 cm) exhibits relatively elevated conductivity, with values reaching 55 S/cm at −2.0 cm. This is consistent with the coarsened yet robust, highly interconnected fibrous architecture shown in Figure 2a, in which encapsulation‐induced bundle aggregation is expected to sustain effective percolative conduction pathways despite the enlarged inter‐bundle voids. A pronounced reduction in conductivity is observed in the vicinity of the junction interface (−0.5 to +0.5 cm), where values decrease to a minimum of 20 S/cm. This localized suppression corresponds spatially to the steep Seebeck coefficient gradient in Figure 3a and is consistent with the structural heterogeneity of the graded transition zone described in Figure 2b, where the coexistence of *p*‐type and *n*‐type morphological features is expected to impede carrier transport and elevate local resistivity. In the *p*‐type region (1.0 to 2.0 cm), the conductivity partially recovers to 27–37 S/cm, remaining intermediate between the *n*‐type region and the junction minimum. Although this recovery reflects the restored network continuity in the homogeneous *p*‐type morphology (Figure 2c), the conductivity values are lower than those in the *n*‐type region, a disparity that may be attributed to differences in intrinsic doping efficiency and carrier concentration between the two semiconducting regions.

The device‐level consequences of this graded transition zone can be estimated quantitatively from the spatial profiles in Figure 3. Because the open‐circuit voltage of a thermoelectric leg with a spatially varying Seebeck coefficient is given by *V* = ∫*S*(*x*)(d*T*/d*x*)d*x*, the effective coefficient of each leg under a smoothly distributed temperature gradient corresponds to the spatial average of *S*(*x*) along the entire leg, including the transition zone. Averaging the measured profile in Figure 3a in this manner yields approximately −40 µV/K for the *n*‐type leg and +16 µV/K for the *p*‐type leg, giving an effective Seebeck difference of ≈56 µV/K per junction—roughly 80% of the 69.0 µV/K expected for an ideal abrupt junction in which each leg retains its region‐averaged coefficient (−45.7 and +23.3 µV/K; see Section 4.2). The measured thermal sensitivity of 45 µV/K per junction pair (Table 2) is consistent with this estimate, the residual reduction being attributable to partial localization of the temperature gradient within the low‐|*S*| transition zone. With respect to internal resistance, integrating the reciprocal of the conductivity profile in Figure 3b over the strip geometry (10 mm width; film thicknesses in Table 1) yields ≈32 Ω per strip, corresponding to ≈65 Ω for the series‐connected device (excluding contact resistance). This film‐only estimate is consistent with the two‐terminal device resistance of 76.2 Ω measured in Section 3.2, which is modestly larger—as expected, since the two‐terminal measurement additionally includes the silver‐paste contact and copper‐interconnect resistances absent from the film‐only estimate—with the residual difference lying within the combined uncertainty of the local film thickness and the position‐discretized conductivity data. The transition zone, although occupying only 25% of the conduction path, contributes approximately one‐third of the strip resistance; nevertheless, relative to a hypothetical abrupt interface in which the conductivity bridges the two plateau values without the localized dip, the graded zone elevates the total internal resistance by only ∼25%. Since the maximum extractable power scales as (Δ*S*_eff_)^2^/*R*_int_, the graded interface limits the attainable power to roughly half of the ideal‐junction value, with the dilution of the effective Seebeck difference—entering quadratically—remaining the larger of the two penalties compared with the added resistance. Conversely, in the open‐circuit sensing mode, the elevated interfacial resistance is largely immaterial, and the sensitivity is governed solely by the effective Seebeck difference. This quantitative assessment underpins the prioritization of interface sharpening as the primary enhancement strategy discussed in Section 4.4.

**TABLE 2:** Voltage response, thermal sensitivity, and response time of the SWCNT *p*–*n* junction devices on different substrates (mean ± standard deviation (SD) over *n* = 3 independently fabricated devices).

Substrate	Peak voltage [mV]	Stable voltage [mV]	Thermal sensitivity [µV/K]	Response time [s]
COP	+0.88 ± 0.30	+0.62 ± 0.22	−9 ± 3	165 ± 6
PI	−0.61 ± 0.07	+0.06 ± 0.05	−37 ± 4	52 ± 11
PEN	−1.07 ± 0.19	−0.01 ± 0.02	−100 ± 17	46 ± 7

*Note*: Values are mean ± standard deviation over *n* = 3 independently fabricated devices per substrate. Device #1 corresponds to the representative trace shown in Figure 4; its peak voltage, stable voltage, and thermal sensitivity equal the previously reported single‐device values. Peak voltage is the extremum of the output voltage after heater activation (*t* = 5 min); response time is the elapsed time from heater activation to that extremum; stable voltage is the mean output over *t* = 12–15 min (the final 3 min of the 15‐min reproducibility window). Thermal sensitivity is defined as the peak output voltage divided by the in‐plane temperature difference across the device; for devices #2 and #3, the in‐plane temperature difference was taken to be identical to that of device #1 on the same substrate, since the three devices were heated under identical conditions, so that the thermal sensitivity scales with the measured peak voltage.

### 3.2 Performance of *p–n* Junction SWCNT Thermoelectric Devices

**FIGURE 4:**
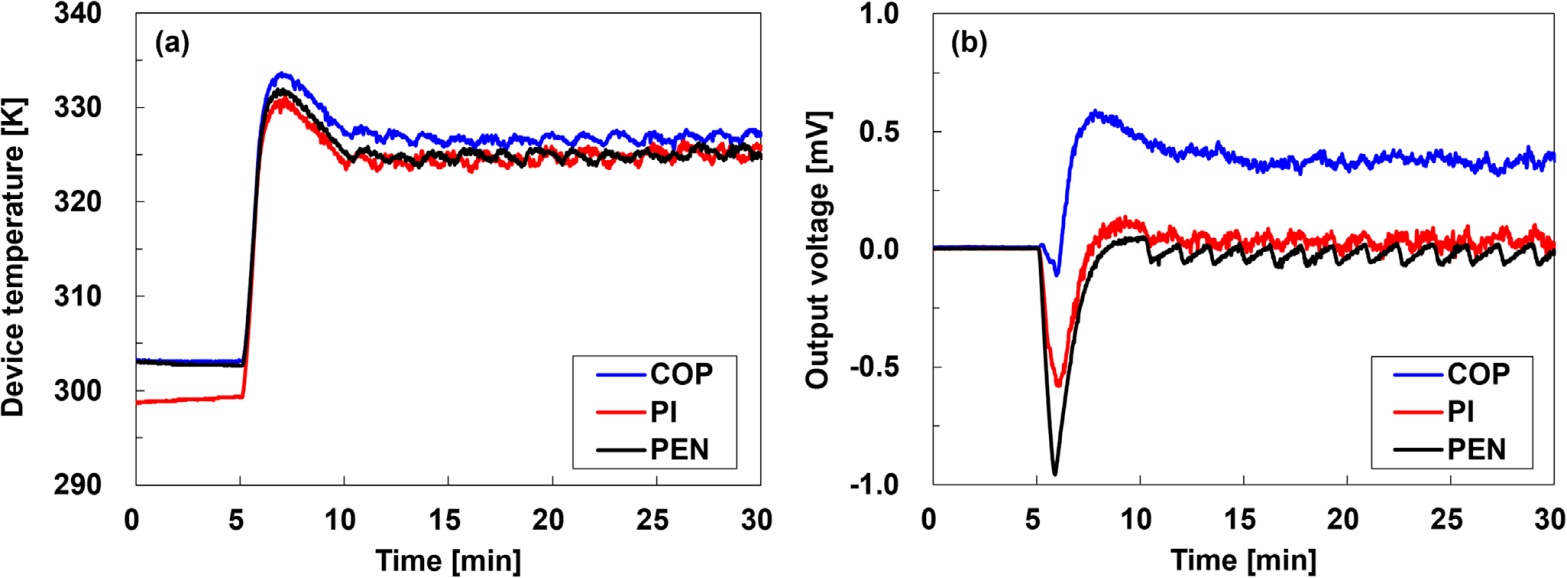
Transient responses of (a) device temperature and (b) output voltage recorded from *p–n* junction SWCNT devices fabricated on PI (red), COP (blue), and PEN (black) substrates upon activation of the hot‐plate heater (target temperature: 325 K). The heater was switched on at approximately 5 min after the start of data acquisition. Note that the three substrates differ in their initial temperatures prior to heater activation (∼299 K for PI; ∼304 K for COP and PEN), reflecting differences in ambient thermal equilibration. In (a), all three devices exhibit a similar qualitative thermal transient profile—a rapid temperature rise with a brief overshoot to ∼333–335 K, followed by relaxation toward substrate‐dependent steady‐state temperatures (∼327–328 K for COP; ∼323–325 K for PI and PEN). In (b), the COP device maintains a positive output voltage throughout: the voltage peaks transiently at +0.59 mV coinciding with the thermal overshoot, before settling to a steady‐state value of +0.38 mV. The PI and PEN devices exhibit immediate negative voltage excursions upon heater activation, reaching peak values of −0.58 and −0.96 mV, respectively. Following the negative peak, the PI device transiently crosses into positive territory before converging toward near‐zero, while the PEN device recovers monotonically toward near‐zero; both devices converge toward near‐zero at thermal equilibrium—the PI device settling slightly positive and the PEN device slightly negative—consistent with the near‐complete collapse of the in‐plane temperature gradient.

Figure 4 presents the transient responses of temperature (a) and output voltage (b) measured from the three *p*–*n* junction SWCNT devices fabricated on PI, COP, and PEN substrates. As shown in Figure 4a, all devices exhibit a similar thermal transient: following heater activation at 5 min, the temperature rises steeply, undergoes a transient overshoot due to the hot‐plate controller, and subsequently relaxes to a steady‐state value near the target temperature of 325 K. A pronounced substrate dependence emerges in the output‐voltage transients, as shown in Figure 4b. All three devices exhibit a brief negative voltage excursion immediately after heater activation; however, the magnitude and subsequent behavior differ markedly among the substrates. The COP‐based device shows only a small initial negative dip, followed by a rapid recovery to a positive output that stabilizes at a positive value. In contrast, the PI‐ and PEN‐based devices exhibit far more pronounced negative excursions with no subsequent recovery to a positive output, instead gradually relaxing toward zero. These results demonstrate that the polarity and magnitude of the transient thermoelectric response depend strongly on substrate identity, indicating that the underlying substrate plays a decisive role in determining the internal temperature distribution and resulting voltage output. To assess reproducibility, three independent devices (*n* = 3) were fabricated and measured on each substrate. The polarity and temporal profile that define each operating mode—sustained positive output for COP and transient negative excursions for PI and PEN—were reproduced in every device without exception, whereas the absolute voltage magnitude showed device‐to‐device variation (largest for COP), which we attribute to batch‐to‐batch variation in the *p*‐type oxygen‐adsorption doping (Section 3.1). Accordingly, the peak voltage, stable voltage, thermal sensitivity, and response time are reported in Table 2 as mean ± standard deviation over the three devices, and the corresponding output‐voltage transients of all three devices on each substrate are provided in Figure S5. These statistics indicate that the substrate‐governed switching is reproducible across the three devices examined rather than specimen‐specific.

**FIGURE 5:**
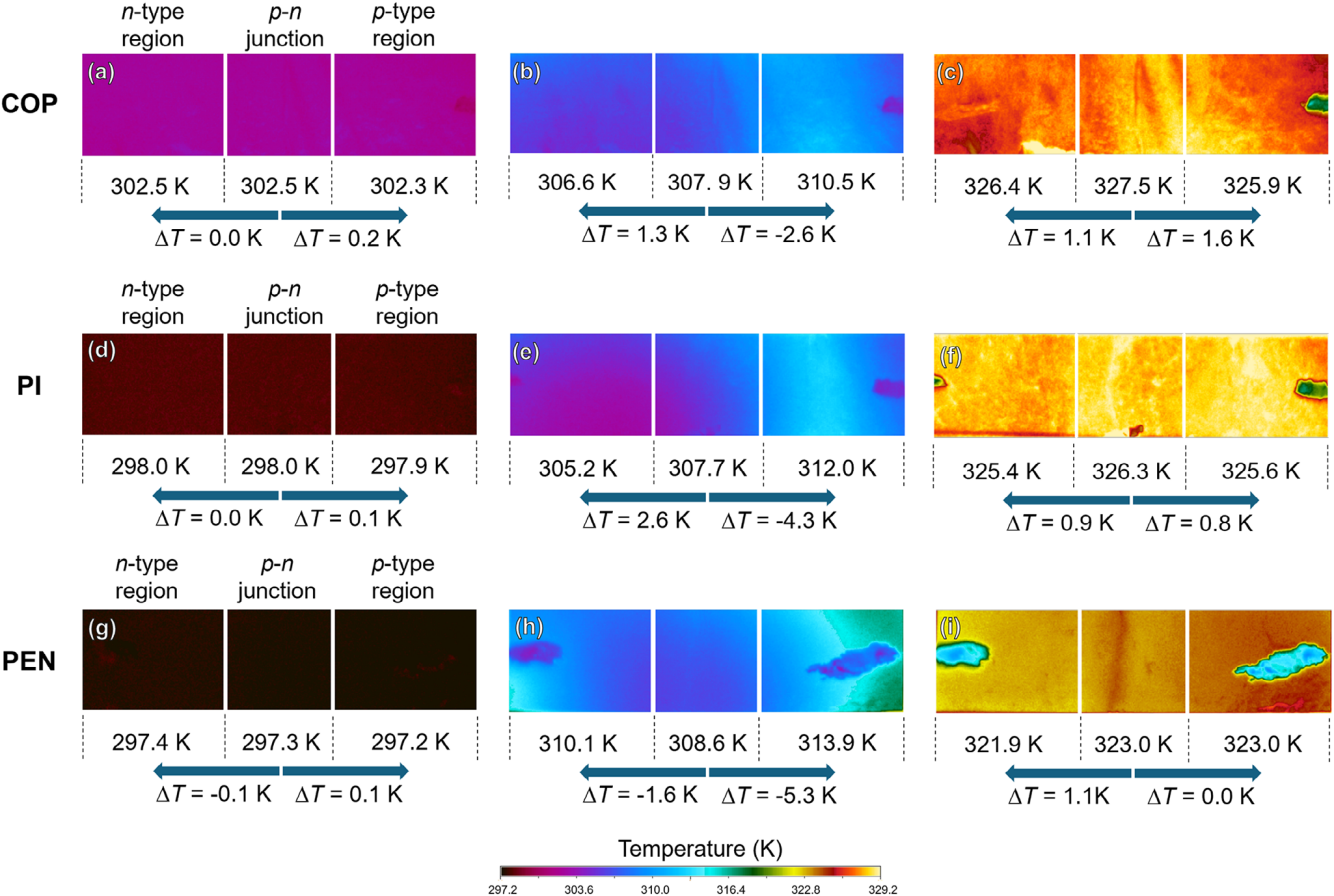
Thermographic images of the device surface recorded for the (a–c) COP‐, (d–f) PI‐, and (g–i) PEN‐based devices at three stages: (a, d, g) heater‐off steady state, (b, e, h) transient immediately following heater activation, and (c, f, i) heater‐on steady state. Within each panel, the left, center, and right zones correspond to the *n*‐type strip, the gap between strips, and the *p*‐type strip, respectively. A single common, fixed false‐color temperature scale (color bar, in K, spanning ∼297–329 K) is applied uniformly to all nine panels, enabling direct visual comparison of the absolute temperature level among substrates and across stages. Because this common scale spans the full ∼32 K range of the three stages, the small (∼1–5 K) in‐plane differences within each device are not resolved by color and are instead annotated numerically on each panel (regional temperatures and the corresponding Δ*T*). Temperature differences are defined as Δ*T* = *T*_junction_ − *T*_outer_end_, so that a positive Δ*T* denotes a junction‐center‐hot gradient favorable for power generation, and a negative Δ*T* denotes an inverted gradient favorable for heat‐flux sensing. For the COP device, the junction region remains warmer than the *n*‐type outer end by Δ*T* = +1.3 K upon activation (b), and a sustained positive gradient (Δ*T* = +1.1 and +1.6 K at the *n*‐type and *p*‐type outer ends, respectively) persists at steady state (c), consistent with the stable positive output voltage in Figure 4b. For the PI device (e), the *p*‐type outer end is hotter than the junction region immediately after activation (Δ*T* = −4.3 K), reflecting transient gradient inversion driven by near‐complete infrared absorption by the substrate surface; this inversion gradually resolves at steady state (f). The PEN device displays the most pronounced inversion (Δ*T* = −5.3 K at the *p*‐type outer end, (h)), attributable to its large thermal inertia, which prolongs surface heat accumulation and produces the largest negative voltage excursion observed (Figure 4(b)); at steady state (i), the residual gradient at the *n*‐type end is Δ*T* = +1.1 K while that at the *p*‐type end is Δ*T* = 0.0 K, and the output voltage converges toward zero.

To quantify and compare these substrate‐dependent voltage responses, the peak voltage, stable voltage, and thermal sensitivity of each device are compiled in Table 2. The voltage and thermal‐sensitivity values quoted in the running text below are those of the representative device (device #1, Figure 4); Table 2 lists the corresponding mean ± standard deviation over the three devices. The peak voltage—defined as the maximum absolute output voltage observed during the abrupt voltage excursion immediately after heater activation—was highest for the PEN‐based device (−0.96 mV), approximately 1.7 times larger than that of the PI‐based device (−0.58 mV), while the COP‐based device exhibited only a brief, small negative excursion before rapidly recovering to a positive peak of +0.59 mV. The stable voltage, defined as the output voltage averaged over *t* = 12–15 min (the final 3 min of the reproducibility measurement window), was +0.38 mV for COP—a value that the extended single‐device run confirms is sustained through *t* = 30 min (Figure 4)—demonstrating its suitability for continuous power generation, whereas PI (+0.03 mV) and PEN (−0.02 mV) effectively converged to zero at thermal equilibrium, consistent with a transient sensing mode. The thermal sensitivity, defined as the peak output voltage divided by the in‐plane temperature difference across the device, was −90 µV/K for PEN, substantially larger in magnitude than −35 µV/K for PI and −6 µV/K for COP. This nearly 15‐fold difference between PEN and COP reflects the contrasting thermal regimes imposed by the respective substrates: PEN's high absorptivity and large thermal inertia amplify the transient voltage signal in response to rapid temperature changes, whereas COP's low absorptivity and small thermal inertia result in only a minimal transient response before the device recovers to stable power generation mode. We note that the thermal sensitivity is, by definition, evaluated at the transient peak; for the COP device this peak corresponds to the small initial negative voltage dip that accompanies the brief overshoot‐driven gradient inversion (negative in‐plane Δ*T* at the peak instant; Figure 5b), so that its thermal sensitivity is negative and small in magnitude even though the steady‐state output—the quantity relevant to the power‐generation mode—is positive. These results show that substrate selection alone can tune device performance across a wide dynamic range, from stable autonomous power generation (COP) to highly sensitive transient heat‐flux detection (PEN).

To quantitatively evaluate the energy‐harvesting capability of the COP‐based device, its internal resistance was measured by the two‐terminal method across the external copper leads at room temperature, yielding *R*_int_ = 76.2 Ω (averaged over six repeated measurements). This configuration was deliberately chosen because it captures the total source resistance presented to an external load—including the SWCNT film, the silver‐paste contacts, and the copper interconnects—which is precisely the quantity governing power delivery. The measured value is modestly larger than the film‐only resistance of ≈65 Ω estimated independently from the spatial conductivity profile in Section 3.1; the modest excess of the measured value over the film‐only estimate is precisely what is expected from the additional silver‐paste contact and copper‐interconnect resistances included in the two‐terminal measurement but absent from the film‐only estimate, and this consistency supports the internal coherence of the transport characterization. Within the Thévenin‐equivalent‐circuit description of a thermoelectric generator, the measured internal resistance and the steady‐state open‐circuit voltage of 0.38 mV (Table 2) fully determine the power‐delivery characteristics: the short‐circuit current is *I*_sc_ = *V*_oc_/*R*_int_ = 5.0 µA, and the maximum output power, delivered at the matched‐load condition *R*_L_ = *R*_int_, is *P*_max_ = *V*_oc_^2^/(4*R*_int_) = 0.47 nW, corresponding to a power density of 0.06 nW/cm^2^ normalized by the total film area (8 cm^2^). The full load‐matching characteristic, calculated as *P*(*R*_L_) = *V*_oc_^2^ *R*_L_/(*R*_L_ + *R*_int_)^2^, is provided in Figure S6. Although these absolute values remain at the proof‐of‐concept level, they establish the baseline metrics against which the enhancement strategies discussed in Section 4.4 can be quantitatively assessed.

Because the SWCNT films possess significantly higher in‐plane thermal conductivities (2.9–3.9 W/(m·K)) than the substrates (0.1–0.2 W/(m·K)) (Table 1), heat absorbed at the film surface is rapidly redistributed laterally within the SWCNT network. Consequently, differences in conductive heat transfer from the hot‐plate cannot account for the substrate‐dependent voltage responses. Instead, the transient behavior reflects substrate‐dependent differences in infrared absorptivity and thermal inertia, which modulate the spatial and temporal evolution of the temperature distribution across the device. A detailed mechanistic interpretation of these substrate‐governed thermal dynamics is presented in Section 4.

Thermographic imaging (Figure 5) provides direct spatial evidence of the substrate‐dependent temperature evolution across all three measurement stages: the heater‐off steady state (Figure 5a,d,g), the transient period immediately after heater activation (Figure 5b,e,h), and the heater‐on steady state at 325 K (Figure 5c,f,i). Note that, to allow direct comparison among the three substrates and across all three measurement stages, a single fixed temperature scale (color bar, ∼297–329 K) is applied uniformly to all nine panels of Figure 5. Because this common scale spans the full ∼32 K range covered by the three stages, the small (∼1–5 K) in‐plane temperature differences within each film are not resolved by color alone; accordingly, the regional temperatures of the *n*‐type region, the junction, and the *p*‐type region, together with the corresponding in‐plane temperature differences, are annotated numerically on each panel. The unified color scale thus enables a direct visual comparison of the absolute temperature level among substrates and stages, while the numerical annotations convey the fine in‐plane gradients that govern the thermoelectric output. Throughout the following discussion, the in‐plane temperature difference is defined as Δ*T* = *T*_junction_ − *T*_outer_end_, so that a positive Δ*T* indicates that the junction center is warmer than the outer end (power‐generation‐favorable gradient), whereas a negative Δ*T* indicates that the outer end is warmer than the junction center (inverted, sensing‐favorable gradient). Because the imaged surface is in all cases the SWCNT film—an optically thick, near‐blackbody emitter (absorbance ∼2.97 at 8.8 µm; Table 1)—a single emissivity (*ε* = 0.95) applies uniformly to all regions, and the spatial temperature differences Δ*T* are insensitive to the absolute emissivity value: any deviation acts as a common‐mode offset that cancels between regions, leaving a residual error in Δ*T* below ∼0.1 K for the gradients reported here. The absolute temperature scale is, in addition, independently anchored by the thermocouple measurement (Figure 4a).

In the heater‐off state, all three devices exhibit a spatially uniform temperature distribution with negligible in‐plane temperature differences across the *n*‐type region, junction, and *p*‐type region, thereby consistent with equivalent initial thermal conditions (Δ*T* ≈ 0 K) across all substrates prior to heater activation (Figure 5a,d,g).

A striking substrate dependence emerges in the transient period immediately after heater activation. For the COP device (Figure 5b), the junction region (307.9 K) is already warmer than the *n*‐type outer end (306.6 K; Δ*T* = +1.3 K), while the *p*‐type outer end (310.5 K; Δ*T* = −2.6 K) is transiently elevated above the junction temperature due to the overshoot dynamics of the hot‐plate controller; nevertheless, the device recovers to fully positive‐gradient operation (Δ*T* > 0 at both ends) as the hot‐plate controller settles. This brief, overshoot‐driven inversion at the *p*‐type outer end—the only segment that momentarily develops a negative in‐plane gradient before the controller settles—accounts for the small initial negative voltage dip exhibited by the COP device in Figure 4b, after which the recovery to a fully junction‐hot gradient drives the sustained positive output. The PI device (Figure 5e) shows a more pronounced asymmetry: the *n*‐type outer end (305.2 K) is cooler than the junction (307.7 K; Δ*T* = +2.6 K), while the *p*‐type outer end (312.0 K) substantially exceeds the junction temperature (Δ*T* = −4.3 K). Most notably, the PEN device (Figure 5h) displays the largest temperature inversion among the three substrates: the *n*‐type outer end (310.1 K) is moderately warmer than the junction (308.6 K; Δ*T* = −1.6 K), while the *p*‐type outer end (313.9 K) substantially exceeds the junction temperature (Δ*T* = −5.3 K). The larger inversion magnitude observed for PEN relative to PI (maximum |Δ*T*| of 5.3 K vs. 4.3 K) is consistent with the greater per‐unit‐area thermal inertia of PEN, which retards heat redistribution from the substrate surface layer and thereby sustains a steeper inverted temperature gradient. These transient inversion patterns correspond directly to the negative voltage excursions in Figure 4b and provide spatial confirmation that the polarity and magnitude of the thermoelectric voltage are governed by the direction and magnitude of the in‐plane temperature gradient.

At thermal steady state, the three substrates produce markedly different spatial temperature profiles. The COP device (Figure 5c) maintains a persistent positive in‐plane gradient, with the junction region (327.5 K) remaining warmer than both outer ends (326.4 and 325.9 K at the *n*‐type and *p*‐type ends, respectively; Δ*T* = +1.1 and +1.6 K, respectively). This sustained gradient drives charge carriers toward the outer ends of the *p*‐ and *n*‐type regions via the Seebeck effect, producing the stable positive output voltage of +0.38 mV observed in Figure 4b. By contrast, the PI device (Figure 5f) reaches near‐thermal equilibrium with only small residual differences (Δ*T* = +0.9 and +0.8 K), resulting in a stable voltage close to zero. Similarly, the PEN device (Figure 5i) retains only small residual in‐plane differences (Δ*T* = +1.1 and 0.0 K), consistent with the near‐zero stable output for that substrate. In contrast to the COP device, where both outer ends remain cooler than the junction and the two legs therefore contribute additively to a net positive output, the residual PEN gradient is both small and one‐sided—appreciable only at the *n*‐type end (Δ*T* = +1.1 K) while having essentially collapsed at the *p*‐type end (Δ*T* = 0.0 K), so that the *p*‐type leg contributes negligibly and the residual single‐leg signal remains close to zero. Collectively, these thermographic images corroborate the substrate‐dependent voltage dynamics shown in Figure 4b and further support the interpretation that substrate infrared absorptivity and thermal inertia are the principal determinants, among the parameters examined, of both the sign and the temporal evolution of the thermoelectric output. Under the unified color scale, all three steady‐state panels (Figure 5c,f,i) sit at the warm, high‐temperature end of the scale, so that the residual in‐plane differences—small relative to the ∼32 K range of the common scale—are most reliably read from the numerical annotations rather than from the color contrast; these annotated values confirm the persistent junction‐hot gradient of the COP device and its near‐complete collapse in the PI and PEN devices.

To further validate the thermoelectric performance of the COP‐based device, the internal temperature difference was estimated from the spatially resolved Seebeck coefficients (Figure 3a) and the steady‐state output voltage (Figure 4b). The average Seebeck coefficients of the *n*‐type and *p*‐type regions were −45.7 and +23.3 µV/K, respectively, giving a per‐junction Seebeck coefficient difference of 69.0 µV/K. The measured steady‐state voltage of 0.38 mV corresponds to 0.19 mV per junction, yielding an estimated internal temperature difference of Δ*T* ≈ 2.8 K. This value is larger than the temperature difference extracted from the thermographic images (∼1.4 K), a trend also reported in previous studies [51]. This discrepancy arises because thermography provides an area‐averaged temperature for each region, which inherently smooths out the local temperature gradients; this roughly twofold discrepancy is therefore physically reasonable and does not compromise the validity of either measurement. These results support that the COP‐based device generates a well‐defined internal temperature gradient sufficient for continuous autonomous thermoelectric power generation.

Finally, the practical robustness of the *p–n* junction device was assessed under both mechanical deformation and repeated thermal cycling. The monolithic *p–n* junction film conformed to a pipe of 32 mm diameter (bending radius ≈ 16 mm) and largely retained the Seebeck coefficients of both the *p*‐type (≈ +50 µV/K) and *n*‐type (≈ −55 µV/K) regions, without appreciable systematic degradation, over 100 bending cycles (Figure S7). Under ten consecutive heating–cooling cycles between ≈ 350 and 375 K—the peak exceeding the 325 K device operating temperature by 50 K—the Seebeck coefficients of both regions were similarly retained (*p*‐type: +51 → +51 µV/K; *n*‐type: −58 → −56 µV/K), suggesting that the DODMAC‐stabilized *n*‐type doping remains stable under the cyclic conditions examined (Figure S8). Because the output voltage in both operating modes is determined by the spatial Seebeck profile of the active film (Equation S3), while the direction of the in‐plane temperature gradient is set by the substrate—whose intrinsic properties are not expected to change appreciably under these tests—the retention of the Seebeck coefficients suggests that the device response is largely maintained in both the power‐generation (COP) and heat‐flux‐sensing (PEN) modes.

## 4 Discussion

The field of flexible SWCNT thermoelectrics has advanced considerably through optimization of the active film itself—refining doping chemistry, tailoring network morphology [52, 53, 54, 55], and engineering junction architecture—yet the role of the supporting substrate has largely been treated as a passive structural variable. This implicit assumption overlooks a critical physical reality: under uniform infrared irradiation, it is not the SWCNT film in isolation but the film–substrate composite that determines the spatial and temporal evolution of the internal temperature gradient, and hence the sign, magnitude, and dynamics of the thermoelectric output. The present results challenge this assumption directly. By holding the active SWCNT layer constant and varying only the substrate, we observed a complete reversal of output‐voltage polarity—from stable positive to transient negative— a finding that is not readily explained by film‐level properties alone and points to a substrate‐centered mechanism. The following sections develop this framework by examining, in turn, how the distinct thermal regimes imposed by COP and by PI/PEN substrates give rise to the two contrasting operating modes identified experimentally.

### 4.1 COP‐Induced Positive Thermoelectric Mode

**FIGURE 6:**
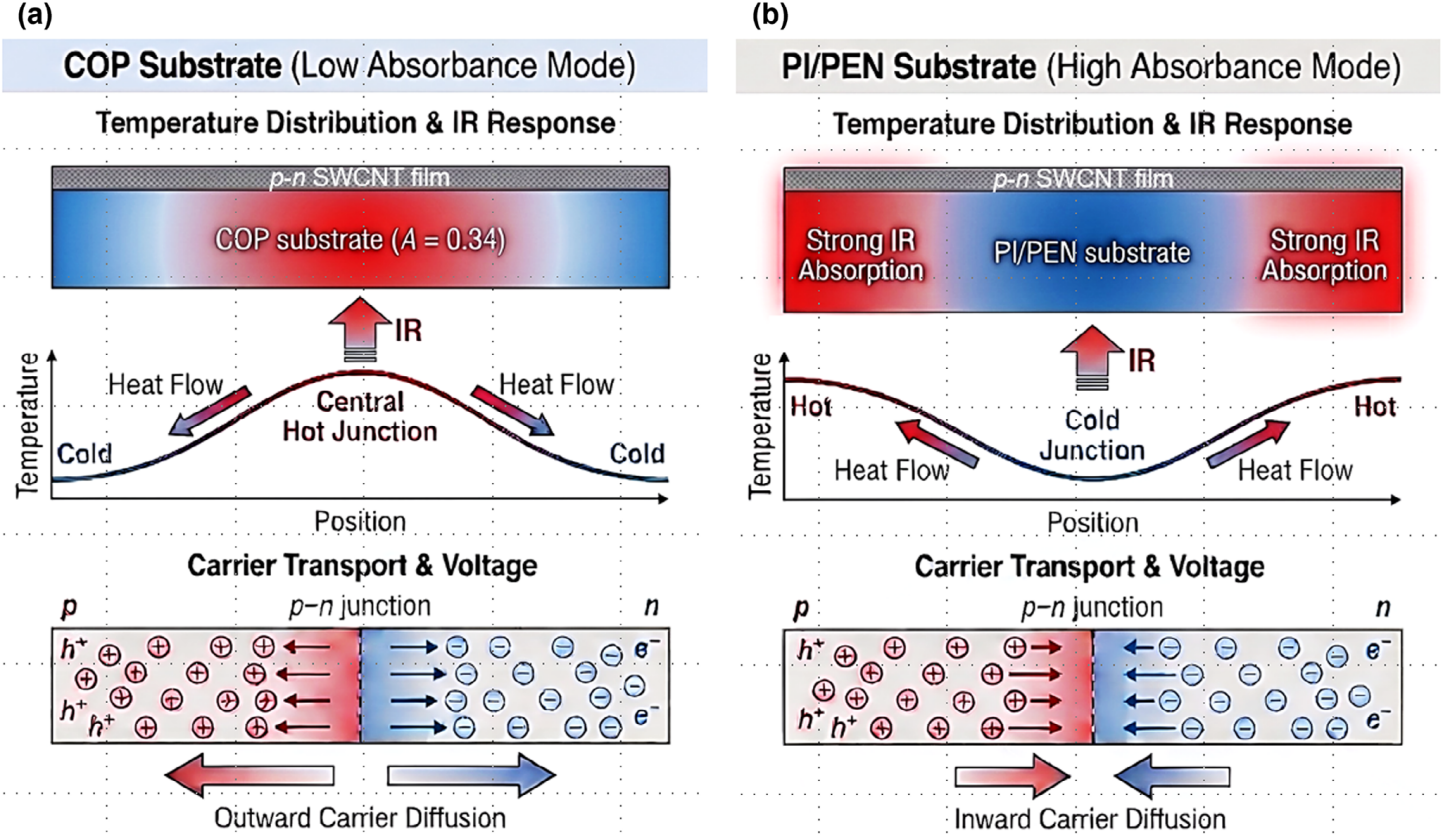
Schematic illustration of the substrate‐dependent voltage generation mechanisms in *p–n* junction SWCNT devices under uniform infrared irradiation incident from below (through the substrate). (a) COP substrate (Low Absorbance Mode): the COP substrate transmits incident IR (transmittance 45.8% at 8.8 µm), allowing the SWCNT film to be heated preferentially. The high in‐plane thermal conductivity of the SWCNT film causes heat to be conducted outward from the junction center toward both outer ends, establishing a central hot‐junction temperature gradient (upper panel). This gradient drives outward carrier diffusion—holes (*h* ^+^) in the *p*‐type region migrate toward the cooler *p*‐type outer end, and electrons (*e* ^−^) in the *n*‐type region migrate toward the cooler *n*‐type outer end (lower panel)—yielding a stable positive output voltage. (b) PI/PEN substrate (High Absorbance Mode): both substrates absorb IR strongly at their surfaces (particularly at the outer end regions), heating the outer ends of the *p–n* junction strips more rapidly than the junction center and inverting the in‐plane temperature gradient to produce a cold junction at the center (upper panel). Heat flows inward from both hot outer ends toward the cooler junction center. This inverted gradient drives inward carrier diffusion—*h* ^+^ and *e* ^−^ both migrating toward the cooler junction center (lower panel)—producing a transient negative output voltage characteristic of heat‐flux sensing mode, sustained by the substrate's high thermal inertia.

Figure 6a illustrates the operating principle of the COP‐based device, showing (i) the formation of an in‐plane temperature gradient within the *p*–*n* patterned SWCNT film (upper panel) and (ii) the corresponding thermally driven carrier diffusion directions in the *p*‐ and *n*‐type regions (lower panel).

The COP‐based device operates under a thermal regime in which the SWCNT film absorbs infrared radiation strongly (absorbance ∼2.97), whereas the COP substrate absorbs only weakly (absorbance 0.34; transmittance 45.8%) (Table 1; original FT‐IR spectra in Figure S2). Infrared radiation is incident from below (through the COP substrate) onto the SWCNT film. As a result, the SWCNT film is heated more intensely than the substrate. Because the SWCNT film exhibits high in‐plane thermal conductivity [2.9–3.9 W/(m·K)] (Table 1), heat absorbed at the junction region is rapidly conducted outward in both directions toward the cooler outer ends of the *p*–*n* junction strips, establishing a stable bell‐shaped in‐plane temperature gradient with the junction region (Central Hot Junction) at a higher temperature than both outer ends (Figure 5c; upper panel of Figure 6a).

This temperature gradient drives thermally induced carrier diffusion: holes (*h* ^+^) in the *p*‐type region migrate from the hot junction center toward the cooler *p*‐type outer end, while electrons (*e* ^−^) in the *n*‐type region migrate toward the cooler *n*‐type outer end — both carrier types undergoing outward diffusion away from the central junction (lower panel of Figure 6a). These opposing but directionally consistent carrier flows generate additive positive potentials at the device terminals, producing the stable positive output voltage observed in Figure 4b. Because the COP substrate transmits rather than absorbs the incident infrared radiation, no transient inversion of the temperature gradient occurs, enabling continuous autonomous power generation under steady‐state uniform heating.

### 4.2 PI/PEN‐Induced Negative Transient Thermoelectric Mode

Figure 6b illustrates the thermal and carrier‐transport behavior characteristic of PI‐ and PEN‐based devices. The upper panel shows that, unlike the COP case, the in‐plane temperature gradient is inverted due to substrate‐dominated surface heating, producing a valley‐shaped (U‐shaped) temperature profile in which the junction center becomes the coldest point (Cold Junction) and both outer ends remain at higher temperatures. The lower panel depicts the corresponding reversal of hole and electron diffusion directions — an inward carrier diffusion — within the *p*‐ and *n*‐type SWCNT regions.

In the PI‐ and PEN‐based devices, both substrates exhibit high infrared absorptivity (PI: 2.84; PEN: 2.71) and negligible transmittance (<0.2%) (Table 1). Infrared radiation is incident from below (through the substrate side), and because the substrates absorb strongly — particularly at both outer end regions, as indicated by the “Strong IR Absorption” zones in Figure 6b — incident infrared radiation is absorbed almost entirely within a thin surface layer of the substrate. Upon heater activation, this near‐surface region is heated more rapidly and intensely than the SWCNT film, causing the outer ends of the *p*–*n* junction strips to reach higher temperatures than the junction center.

This substrate‐driven near‐surface heating reverses the in‐plane temperature gradient relative to the COP case: the outer ends become hotter than the junction region, and heat flows inward from both ends toward the cooler junction center (Figure 5e,h; upper panel of Figure 6b). Under this inverted gradient, holes (*h* ^+^) in the p‐type region diffuse from the hot outer end toward the cooler junction center (inward, →), while electrons (*e* ^−^) in the *n*‐type region similarly diffuse toward the junction center from the *n*‐type outer end (inward, ←) — both carrier types undergoing inward diffusion converging on the central *p*–*n* junction, in directions opposite to those observed in the COP device (lower panel of Figure 6b). These reversed carrier flows generate negative potentials at the device terminals, producing the transient negative voltage excursions observed in Figure 4b.

The magnitude and duration of the negative voltage response are governed by substrate thermal inertia. PEN possesses a 2.4‐fold greater per‐unit‐area thermal inertia than PI due to its larger thickness (250 vs. 125 µm) and higher specific heat [1.3 vs. 1.1 J/(g·K)] (Table 1). This greater thermal inertia slows the redistribution of surface‐absorbed heat, prolonging the inverted‐gradient regime and amplifying the negative voltage excursion to −0.96 mV — the largest among the three substrates. Although PI and PEN also differ slightly in infrared absorbance (2.84 vs. 2.71), this difference is physically negligible: both values correspond to near‐complete infrared absorption, with transmittances of only 0.15% and 0.20%, respectively (Table 1). The residual transmitted fraction is therefore insufficient to produce a measurable difference in photothermal heating intensity between the two substrates. Consequently, the differential voltage response between PI and PEN—a 1.7‐fold difference in peak voltage magnitude (−0.58 vs. −0.96 mV) and a 2.6‐fold difference in thermal sensitivity (−35 vs. −90 µV/K)— is therefore unlikely to arise from differences in infrared absorptivity and is instead attributed primarily to the 2.4‐fold difference in per‐unit‐area thermal inertia.

### 4.3 Quantitative Validation by a Transient Thermal Model

**FIGURE 7:**
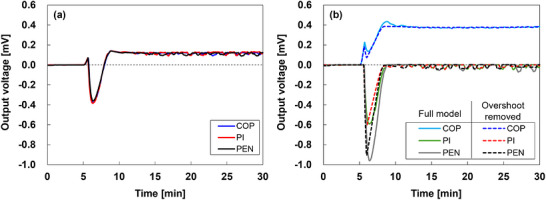
Decoupling of the heat‐transfer pathways by the transient thermal model. (a) When the three substrates are assigned identical optical properties—while contact conduction, convection, and the measured hot‐plate overshoot are left unchanged—the polarity reversal disappears, and the COP, PI, and PEN devices collapse onto a single small response, indicating that the substrate‐dependent switching is governed primarily by the contrast of substrate infrared absorptivity. (b) Removing the hot‐plate overshoot from the driving temperature (dashed curves) leaves the full‐model response (solid curves) essentially unchanged in sign and magnitude, indicating that the overshoot modulates rather than causes the transient response. The full model formulation, parameters, and the model‐versus‐experiment comparison are provided in Figures S9 and S10.

To corroborate the qualitative mechanism of Figure 6 and to disentangle the heat‐transfer pathways inherent to the hot‐plate measurement—contact conduction, air convection, hot‐plate overshoot, and infrared absorption—we developed a reduced‐order transient thermal model of the in‐plane temperature distribution along a single *p–n* strip, driven directly by the measured device temperature of Figure 4a and converted to output voltage through the measured Seebeck profile of Figure 3a (full formulation, parameters, and computed temperature profiles in Figures S9 and S10). Following a one‐time calibration to three measured voltages, the model reproduces, without further adjustment, the sign, magnitude, and temporal evolution of the output voltage of all three devices—the sustained positive response of COP and the transient negative excursions of PI and PEN that relax toward zero at steady state. More importantly, the model helps disentangle the contributions of the substrate‐dependent switching (Figure 7): when the three substrates are assigned identical optical properties, while the contact conduction, convection, and measured overshoot are left unchanged, the polarity reversal no longer appears and the three devices collapse onto a single response (Figure 7a); conversely, removing the hot‐plate overshoot from the driving temperature leaves the sign and approximate magnitude of every response unchanged (Figure 7b). In both numerical experiments, all calibrated model parameters listed in Table S2—the two photothermal gain coefficients, the thermal‐inertia exponent, the film properties, and the substrate thermal inertia—were held fixed at their calibrated values; the matched‐optics experiment of Figure 7a altered only the substrate transmittance and absorptance, equalizing them across the three devices while leaving the measured driving temperature (and hence the contact‐conduction, convection, and overshoot contributions) unchanged, whereas the overshoot‐removal experiment of Figure 7b altered only the driving temperature, replacing it with its slowly varying trend while keeping all optical and thermal parameters fixed. These controlled numerical experiments indicate that, within the model, the polarity reversal is driven primarily by the contrast of substrate infrared absorptivity —not by the conductive, convective, or interfacial contributions common to all devices (including the identical adhesive layer), nor in the hot‐plate overshoot—thereby providing quantitative support for the substrate‐absorptivity and thermal‐inertia mechanism developed above.

### 4.4 Broader Implications and Future Directions

The contrast between COP and PEN substrates supports the central conclusion of this study: the substrate is not a passive mechanical support but an active thermal component that largely governs the functional mode of the SWCNT *p*–*n* junction architecture. Depending primarily on substrate absorptivity and thermal inertia, the same device architecture can operate either as a continuous power generator (COP substrate) or as a transient heat‐flux sensor (PEN substrate). This substrate‐governed bifunctionality enables multiple IoT applications without altering the active SWCNT layer or circuit configuration.

Building on this conclusion, several engineering pathways can further enhance device performance—particularly for autonomous power generation on COP substrates. Although the COP‐based device produces a steady‐state voltage of 0.38 mV, additional enhancement is required for direct integration with commercial boost converters, which typically require input voltages near 20 mV. Three complementary strategies are envisioned. First, sharpening the *p*–*n* junction interface to suppress interdiffusion of n‐type and p‐type dispersions would increase the effective Seebeck coefficient difference and raise the voltage per unit temperature gradient. Second, computational thermal–fluid optimization may identify device geometries that maximize the internal temperature gradient under uniform heating. Third, replacing the Kapton tape adhesive layer—currently used to bond the SWCNT film to the substrate—with an infrared‐transparent bonding layer would reduce parasitic absorption at the film–substrate interface and thereby strengthen the photothermal driving force. In addition to these device‐level refinements, the output voltage can be scaled by serializing junction pairs: because both the open‐circuit voltage and the internal resistance increase linearly with the number of series‐connected pairs N, the maximum output power also scales linearly with N, and approximately 10553 pairs—achievable by extending the same sequential vacuum filtration and dicing process—would reach the ∼20 mV input threshold of commercial boost converters. The fabrication of such multi‐junction arrays, together with capacitor‐charging demonstrations, is therefore identified as a concrete next step toward practical self‐powered operation.

Conversely, the PEN‐based device leverages its high thermal inertia and strong infrared absorptivity to function as a highly responsive transient heat‐flux sensor. The prototype, featuring two serialized *p*–*n* junctions, demonstrates a total thermal sensitivity of −90 µV/K, which corresponds to −45 µV/K per single *p*–*n* junction pair (values for the representative device #1; the corresponding mean ± standard deviation over the three devices is given in Table 2). This value is comparable to the standard Seebeck coefficient of K‐type thermocouples (41 µV/K) [56], yet remains below that of state‐of‐the‐art inorganic thin‐film junctions, which have recently achieved 272 µV/K per single junction pair using BiSb–Sb_2_Te_3_ films [57]. To bridge this performance gap, two parallel strategies are proposed: (i) optimizing SWCNT network density and morphology to enhance the intrinsic Seebeck coefficient of the active layer [58], and (ii) engineering the substrate by reducing PEN thickness or introducing a micro‐porous structure to decrease thermal mass, thereby amplifying the instantaneous temperature differential across the *p*–*n* junction and approaching the benchmarks set by established inorganic thermoelectric materials. In parallel, the intrinsic thermoelectric figure of merit of the active layer may be further improved by phonon‐scattering strategies analogous to those developed for inorganic systems, such as the dopant‐induced lattice phonon scattering recently demonstrated to substantially lower the thermal conductivity of SnSe_2_ [59].

Beyond these device‐level refinements, the substrate‐engineering concept introduced here points toward a broader design principle: the deliberate spatial patterning of substrate thermal properties within a single device. The transient thermoelectric response is governed locally by the product of the Seebeck coefficient and the instantaneous temperature gradient. Because that gradient depends on the position‐dependent interplay between infrared absorptivity and thermal inertia, regions of intentionally contrasting thermal inertia within the same substrate could generate voltage signals that differ not only in magnitude but also in polarity and temporal profile. This spatial selectivity opens the possibility of monolithic SWCNT devices capable of spatially multiplexed sensing or thermally encoded logic operations—a concept that draws parallels with emerging phononic and radiative thermal logic architectures [60, 61, 62]. Realizing such devices will require advances in substrate micropatterning and multi‐channel readout electronics, but the physical principles demonstrated here provide a clear foundation for these developments.

## 5 Conclusions

We show that substrate engineering serves as a key design parameter for flexible SWCNT thermoelectric devices operating under spatially uniform infrared irradiation. By systematically comparing three commercially available polymer substrates—COP, PI, and PEN—we have shown that the sign, magnitude, and temporal evolution of the output voltage are governed not by the active SWCNT film alone, but by two substrate‐specific thermal parameters: infrared absorptivity at the peak blackbody emission wavelength (∼8.8 µm) relevant to near‐room‐temperature operation (∼325 K), and per‐unit‐area thermal inertia (the product of specific heat capacity and areal mass).

COP, with its exceptionally low infrared absorbance (0.34) and high transmittance (45.8% at 8.8 µm), allows incident radiation to pass through the substrate and heat the SWCNT film preferentially, thereby sustaining a stable in‐plane temperature gradient with the *p*–*n* junction center as the hot point. This enables continuous autonomous power generation, delivering a stable positive output voltage of +0.38 mV at steady state. By contrast, PI and PEN—both of which exhibit near‐complete infrared absorption (absorbance 2.84 and 2.71, respectively)—absorb radiation predominantly within the substrate itself, transiently inverting the in‐plane temperature gradient upon heater activation and producing negative voltage excursions of −0.58 and −0.96 mV, respectively. The larger response of PEN is attributed to its 2.4‐fold greater per‐unit‐area thermal inertia relative to PI, which retards transverse heat redistribution and prolongs the inverted‐gradient regime, making PEN particularly well‐suited for high‐sensitivity heat‐flux sensing. Thermographic imaging directly corroborates these gradient dynamics at all three measurement stages.

These findings establish a two‐parameter substrate‐engineering framework—infrared absorptivity and thermal inertia—that, for the present device structure and testing conditions, produces a substrate‐dependent transition of a single SWCNT *p*–*n* junction architecture between continuous power generation and high‐sensitivity heat‐flux sensing, governed primarily by the choice of substrate while the active film and circuit configuration are held constant. This substrate‐dependent bifunctionality offers a straightforward route to reduced fabrication complexity and opens a rational design pathway for versatile thermoelectric systems targeting next‐generation IoT applications.

## Author Contributions

M.T. conceived the idea and discussed and analyzed the data. R.T. designed and performed most of the experiments and drafted the manuscript. H.N. and S.O. assisted in constructing the apparatus for property measurements. R.T. and M.T. wrote and edited the manuscript. All the authors commented on the manuscript and agreed with the results and conclusions.

## Funding

This study was partially supported by the Hiratsuka City‐Industry (Kanto Yakin Kogyo Co., Ltd.) Academia Joint Research Commercialization Support Grant.

## Conflicts of Interest

The authors declare no conflicts of interest.

## Supporting information




**Supporting File**: advs76889‐sup‐0001‐SuppMat.docx.

## Data Availability

The data supporting the findings of this study are available from the corresponding author upon reasonable request.
