# Supplementary material for: Substrate Engineering of SWCNT p–n Junctions for Dual‐Mode Power Generation and Heat‐Flux Sensing
Source: Adv Sci (Weinh). 2026 Jul 30:e76889. Online ahead of print. doi: 10.1002/advs.76889 (PMC13423488; doi:10.1002/advs.76889)
Supplement: Supplementary file 1 — Supporting File: advs76889‐sup‐0001‐SuppMat.docx. [file ADVS-9999-e76889-s001.docx]

**Electronic Supplementary Information**

for

**Substrate Engineering of SWCNT *p*–*n* Junctions for Dual-Mode Power Generation and Heat-Flux Sensing**

Ryota Tamai^1^, Hiroto Nakayama^1^, Shuya Ochiai^1^, Masayuki Takashiri^1,a)^

^1^Department of Materials Science, Tokai University, Hiratsuka, Kanagawa 259-1292, Japan.

^a)^ Author to whom correspondence should be addressed: takashiri@tokai.ac.jp

**S1. Bundle-diameter distribution analysis**


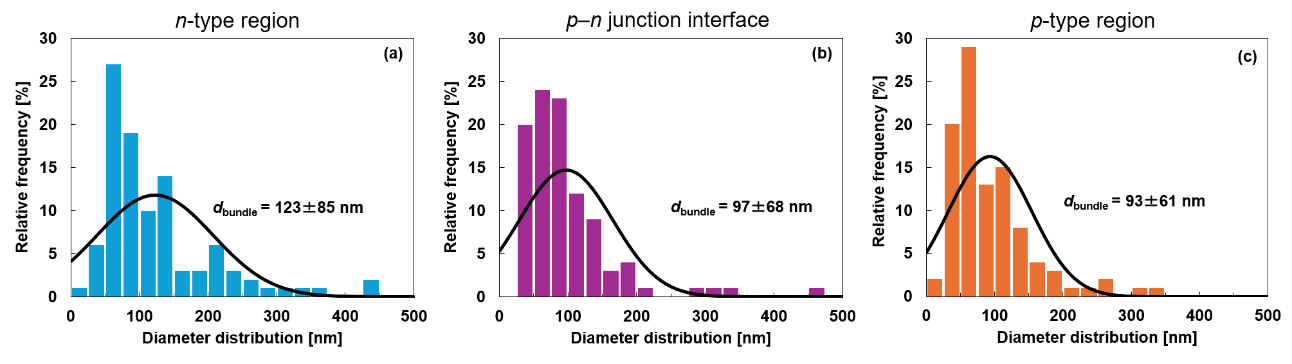


Figure S1. Bundle-diameter distributions of the SWCNT film surface in the (a) *n*-type region, (b) *p–n* junction interface, and (c) *p*-type region, obtained from the SEM micrographs in Figure 2 by image analysis (ImageJ). For each region, 100 bundles were measured and grouped into 25-nm classes; bars show the relative frequency, the solid curve is a log-normal fit, and the dashed line marks the distribution mean. The mean, standard deviation, and median (*n* = 100) are indicated in each panel. The distributions are right-skewed in all three regions, and the mean bundle diameter decreases from the *n*-type region (123 ± 85 nm) through the *p–n* junction interface (97 ± 68 nm) to the *p*-type region (93 ± 61 nm), confirming the morphological trend described in the main text.

The bundle-diameter distributions of the three regions were determined from the surface SEM micrographs shown in Figure 2 using the image-analysis software ImageJ. After spatial calibration using the embedded scale bar in each micrograph, 100 bundles per region were randomly sampled across the field of view; for each bundle, the diameter was defined as its full width, measured along a straight line drawn perpendicular to the local bundle axis at the bundle midpoint. To minimize sampling bias, bundles were selected without regard to their apparent thickness, and partially overlapping or out-of-focus bundles were excluded from the measurement.

The measured diameters were grouped into 25-nm classes, and the relative frequency, mean, standard deviation, and median were computed for each region (Fig. S1). In all three regions the distributions are right-skewed, with the dominant bundle population located in the 50–150 nm range and a tail extending toward larger diameters; accordingly, a log-normal function describes the distributions well and is overlaid in Fig. S1. The mean bundle diameter is largest in the *n*-type region (123 ± 85 nm), intermediate-to-fine at the *p–n* junction interface (97 ± 68 nm), and smallest in the *p*-type region (93 ± 61 nm). The difference between the *n*-type region and the other two regions is statistically meaningful, whereas the junction interface and the *p*-type region are essentially indistinguishable in their mean diameter, consistent with the SEM observation that the junction morphology approaches that of the *p*-type region. This region-dependent coarsening is attributed to the encapsulation of individual SWCNT bundles by DODMAC molecules in the *n*-type region, which promotes inter-bundle aggregation.

**
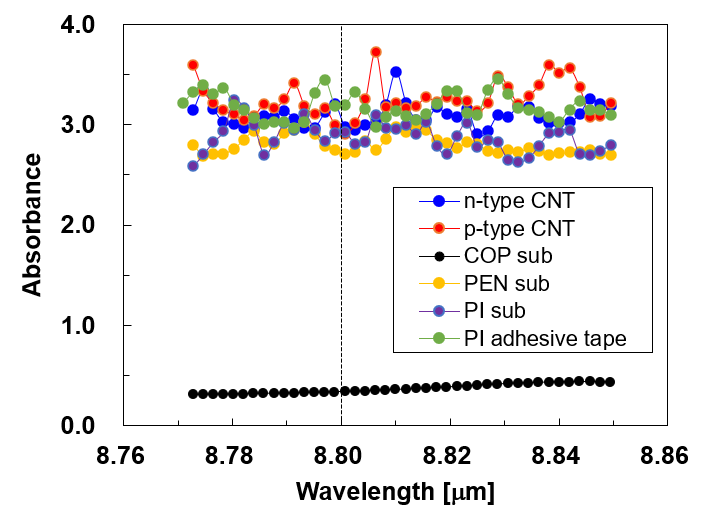
S2. Infrared optical properties of the films, substrates, and adhesive layer**

Figure S2. Fourier-transform infrared (FT-IR) absorbance spectra of the *p*-type and *n*-type SWCNT films, the three substrates (COP, PI, and PEN), and the Kapton double-sided adhesive tape, recorded with a narrowed wavenumber interval in the vicinity of 8.8 μm to ensure quantitative accuracy at this wavelength. The dashed vertical line marks 8.8 μm, which is close to the peak blackbody emission wavelength of the hot plate at the operating temperature of 325 K (Wien’s displacement law, *λ*_max_ = 2898 μm·K/325 K ≈ 8.9 μm), at which the absorbance and transmittance values listed in Table 1 (main text) and Table S1 are extracted.

Figure S2 presents the original FT-IR absorbance spectra of the SWCNT films, the three substrates, and the Kapton double-sided adhesive tape in the vicinity of 8.8 μm, from which the absorbance and transmittance values reported in Table 1 (main text) and Table S1 are directly extracted. The wavelength of 8.8 μm is close to the peak of the blackbody radiation emitted by the hot plate at the operating temperature of 325 K (Wien’s displacement law); the optical contrast among the substrates at this wavelength is therefore the dominant factor governing the photothermal response of the devices. Accordingly, the measurements were performed with a deliberately narrowed wavenumber interval around 8.8 μm to ensure the quantitative accuracy of the extracted values. The spectra directly confirm the optical contrast invoked throughout the main text: the COP substrate exhibits a markedly lower absorbance (≈0.34) than the PI and PEN substrates, both of which show near-complete absorption (absorbance 2.7–3.0, corresponding to a transmittance below 0.2%), while the SWCNT films and the adhesive tape are strongly absorbing (absorbance ≈3.0 or higher). The spectrum of the adhesive tape additionally characterizes the parasitic infrared absorption of the bonding layer discussed in Section 4.4 of the main text.

**S3. Mechanical properties of SWCNT films**


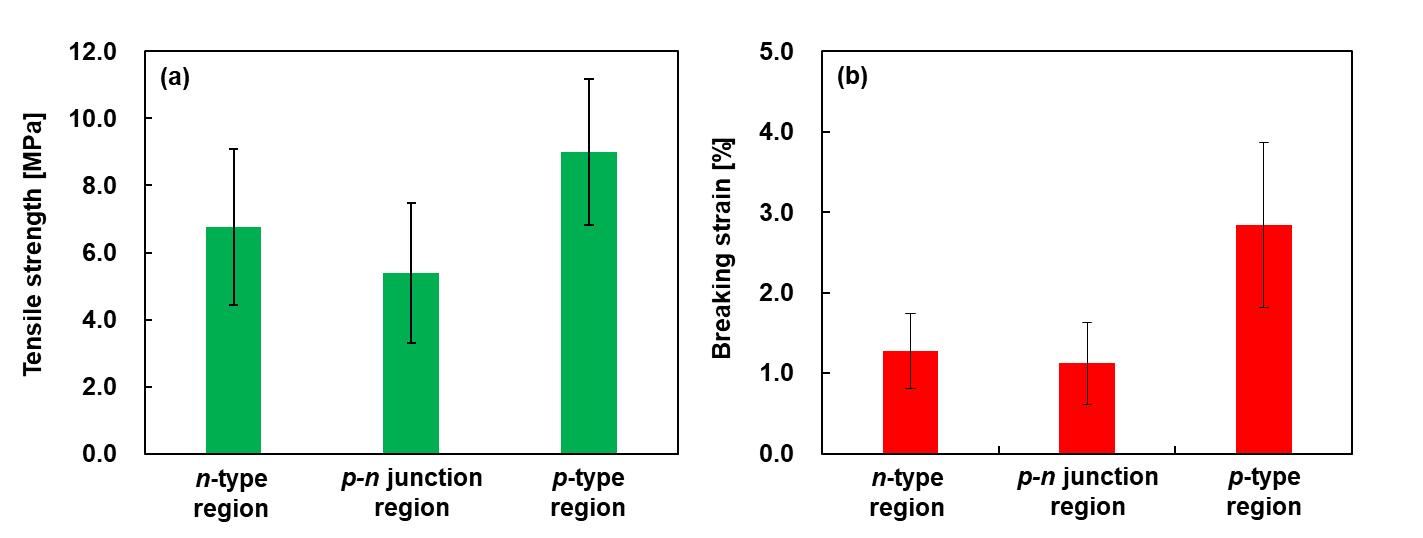
Figure S3. Mechanical properties of SWCNT films in the *n*-type, *p–n* junction, and *p*-type regions. (a) Tensile strength and (b) breaking strain measured at approximately 300 K. Error bars represent the standard deviation of five specimens per region.

Figure S3 presents the tensile strength and breaking strain of the SWCNT films measured in the *n*-type, *p–n* junction, and *p*-type regions. As shown in Fig. S3(a), the tensile strength of the *p*-type region (~8.9 MPa) is the highest among the three regions, exceeding that of the *n*-type region (~6.7 MPa) and the *p–n* junction region (~5.4 MPa). This trend is consistent with the dense, homogeneous network morphology of the *p*-type region (Fig. 2(c)), in which the high packing density of SWCNT bundles with minimal inter-bundle voids maximizes load-bearing cross-sectional area and network continuity. The relatively lower tensile strength of the *n*-type region is attributed to the enlarged inter-bundle voids induced by DODMAC encapsulation (Fig. 2(a)), which reduces the effective load-transfer pathways across the network. The *p–n* junction region exhibits the lowest tensile strength, reflecting the structural heterogeneity of the graded transition zone in which the coexistence of *p*-type and *n*-type morphological features disrupts the network uniformity (Fig. 2(b)).

A similar trend is observed in the breaking strain (Fig. S3(b)): the *p*-type region shows the highest breaking strain (~2.8%), whereas both the *n*-type (~1.3%) and *p–n* junction (~1.1%) regions exhibit substantially lower values. Despite these region-dependent differences, all three regions retain sufficient mechanical flexibility for integration into flexible thermoelectric devices, and the overall mechanical performance of the monolithic *p–n* junction SWCNT film is adequate for the substrate-bonded device architecture described in this study.

**S4. Batch-to-batch reproducibility of the graded *p*–*n* junction interface**


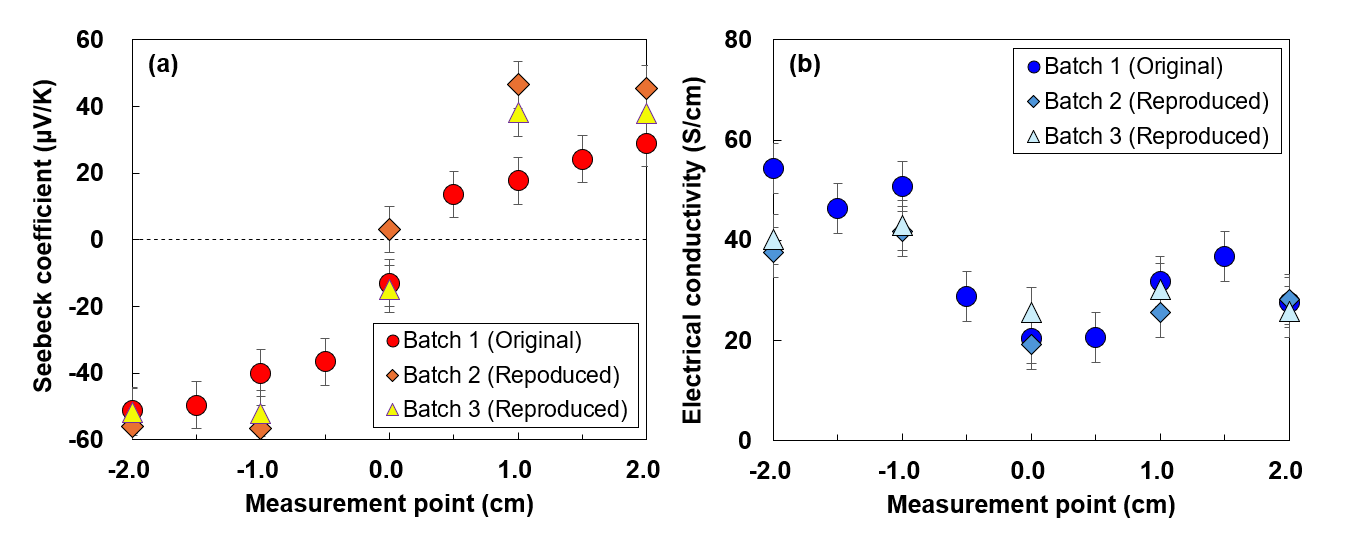


Figure S4. Batch-to-batch reproducibility of the graded *p–n* junction interface. Spatial profiles of (a) the Seebeck coefficient and (b) the electrical conductivity for two additional monolithic *p–n* junction SWCNT films (Batches 2 and 3), fabricated in independent batches under exactly the same sequential vacuum filtration and masking protocol as the original film (Batch 1, reproduced from Fig. 3). The additional batches were measured at five positions (10-mm intervals) along the longitudinal axis, whereas the original film was measured at nine positions (5-mm intervals). Both additional films reproduce the characteristic features of the graded interface: the Seebeck coefficient varies monotonically from −56 and −51 μV/K in the *n*-type region (−2.0 cm) to +45 and +38 μV/K in the *p*-type region (+2.0 cm), with the polarity crossover located at the geometric junction position (+3 and −15 μV/K at 0 cm, compared with −13 μV/K for the original film), and the electrical conductivity exhibits the same localized minimum at the junction (19 and 26 S/cm at 0 cm, compared with 20 S/cm for the original film). The *n*-type plateau values (−51 to −57 μV/K) agree closely among the three batches, reflecting the stable DODMAC doping, whereas the somewhat higher *p*-type plateau values of the additional batches (+38 to +47 μV/K versus +29 μV/K) are attributed to batch-to-batch variation in the degree of atmospheric oxygen-adsorption doping, which does not affect the graded character of the interface itself.

**
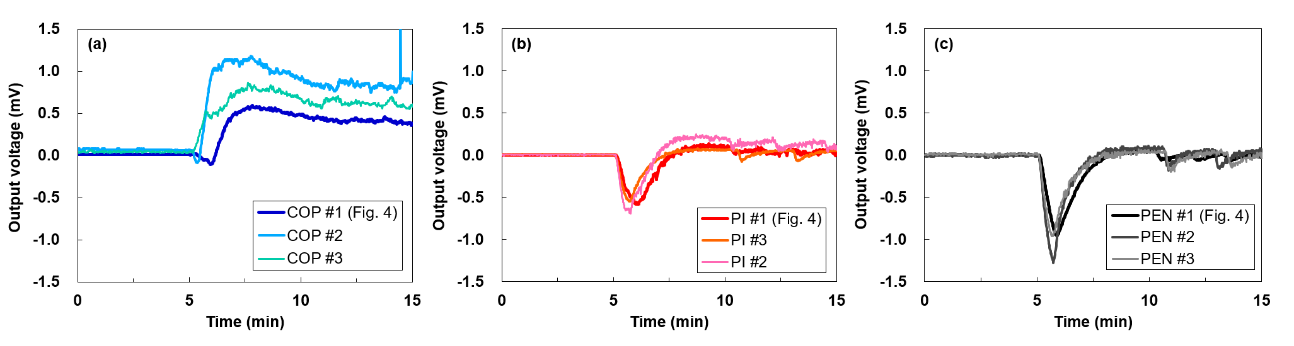
S5. Device-to-device reproducibility of the transient output-voltage response**

Figure S5. Device-to-device reproducibility of the transient output-voltage response. Output voltage of three independently fabricated *p–n* junction SWCNT devices (#1–#3) on (a) COP, (b) PI, and (c) PEN substrates, recorded under the same uniform-heating protocol as in Fig. 4 (hot-plate heater switched on at *t* = 5 min; target temperature 325 K). In each panel, device #1 (bold trace) is the representative device whose response is shown in Fig. 4. The isolated spike near 14.5 min in COP #2 [panel (a)] is a single-point measurement artifact and was excluded from the stable-voltage average reported in Table 2.

Figure S5 presents the output-voltage transients of three independently fabricated devices on each substrate. On all three substrates, the polarity and temporal profile of the response are reproduced across the three devices: the COP devices [Fig. S5(a)] consistently recover to a sustained positive output after a small initial negative dip, whereas the PI [Fig. S5(b)] and PEN [Fig. S5(c)] devices consistently exhibit a pronounced transient negative excursion that relaxes toward zero. The absolute magnitude of the response varies from device to device—most noticeably for COP, whose steady-state output ranges from +0.37 to +0.84 mV—which we attribute to batch-to-batch variation in the *p*-type oxygen-adsorption doping discussed in Section 3.1 and documented at the material level in Fig. S4. The statistical summary (mean ± SD, *n* = 3) of the peak voltage, stable voltage, thermal sensitivity, and response time derived from these transients is given in Table 2 of the main text.

**S6. Load-matching characteristics of the COP-based device**


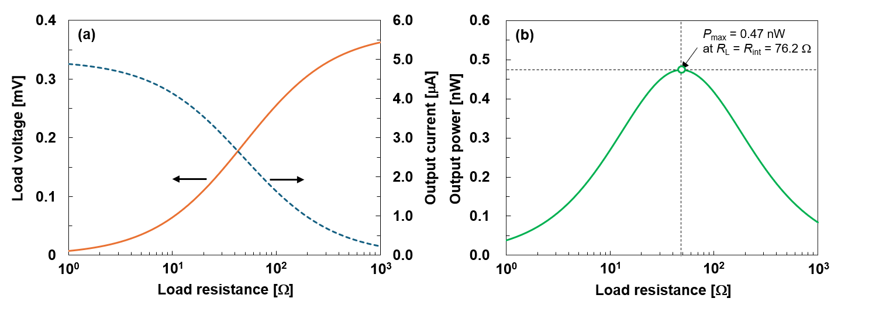
Figure S6. Load-matching characteristics of the COP-based *p–n* junction SWCNT device under the steady-state heating condition, calculated from the two measured parameters: the steady-state open-circuit voltage *V*_oc_ = 0.38 mV (Table 2) and the internal resistance *R*_int_ = 76.2 Ω, determined by the two-terminal method across the external copper leads (averaged over six repeated measurements). (a) Load voltage *V*_L_ = *V*_oc_ *R*_L_/(*R*_L_ + *R*_int_) and output current *I* = *V*_oc_/(*R*_L_ + *R*_int_) as functions of the load resistance *R*_L_; the short-circuit current is *I*_sc_ = 5.0 μA. (b) Output power *P*(*R*_L_) = *V*_oc_² *R*_L_/(*R*_L_ + *R*_int_)^2^, exhibiting the maximum *P*_max_ = *V*_oc_^2^/(4*R*_int_) = 0.47 nW at the matched-load condition *R*_L_ = *R*_int_ (vertical dotted line), corresponding to a power density of 0.06 nW/cm² normalized by the total film area (8 cm²).

Within the Thévenin-equivalent-circuit description of a thermoelectric generator, the load-matching characteristics shown in Fig. S6 follow directly from the two measured parameters (*V*_oc_ and *R*_int_) and quantify the energy-harvesting capability of the present proof-of-concept device.

**S7. Mechanical and thermal-cycling durability of the *p–n* junction device**


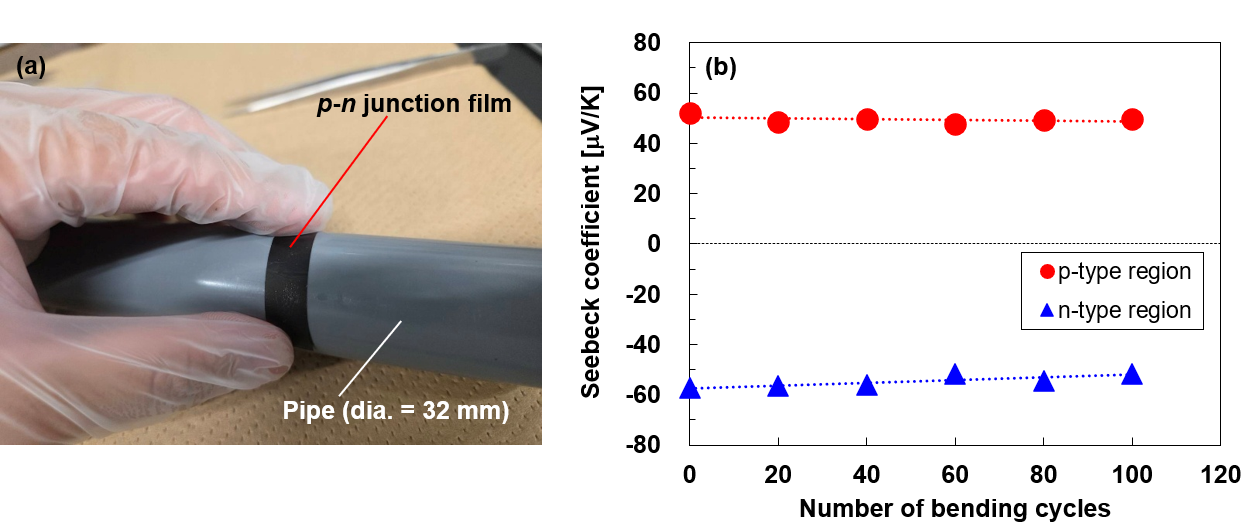
Because the target IoT applications require the device to tolerate mechanical deformation and repeated thermal excursions, the durability of the monolithic *p–n* junction SWCNT film was evaluated under two protocols: repeated bending and repeated heating–cooling. In both protocols, durability was assessed through the Seebeck coefficients of the *p*-type and *n*-type regions, which govern the device output in both operating modes through *V* = ∫*S*(*x*)(d*T*/d*x*)d*x* (Eq. S3); the substrate, which selects the direction of the in-plane temperature gradient and hence the operating mode, is a commercial polymer film whose optical and thermal properties are not expected to be appreciably altered by these tests.

Figure S7. Mechanical bending durability of the *p–n* junction SWCNT film. (a) Photograph of the film conformed to a cylindrical pipe of 32 mm diameter (bending radius ≈ 16 mm). (b) Seebeck coefficients of the *p*-type and *n*-type regions as a function of the number of bending cycles, up to 100 cycles. Both regions largely retain their sign and magnitude—*p*-type at ≈ +50 μV/K and *n*-type at ≈ −55 μV/K—throughout the test, with variations within ≈ 10% and no appreciable systematic degradation.

For the bending test, the film was repeatedly conformed to and released from a pipe of 32 mm diameter (bending radius ≈ 16 mm), and the Seebeck coefficients of the two regions were measured every 20 cycles up to 100 cycles. As shown in Fig. S7(b), neither region exhibits an appreciable systematic change: the *p*-type Seebeck coefficient remains close to ≈ +50 μV/K, and the *n*-type value remains close to ≈ −55 μV/K, the small scatter lying within the measurement reproducibility. The retention of both polarities and magnitudes indicates that the SWCNT network and the *p–n* junction tolerate repeated bending at a curvature representative of practical pipe- or surface-mounted deployment.

Figure S8. Thermal-cycling durability of the *p–n* junction SWCNT film. (a) Substrate temperature during ten consecutive heating–cooling cycles: each cycle consists of rapid heating to ≈ 375 K followed by cooling to ≈ 350 K, over a total of ≈ 4600 s. The peak temperature of 375 K exceeds the 325 K device operating temperature by 50 K, providing a relatively demanding thermal stress. (b) Seebeck coefficients of the *p*-type and *n*-type regions measured before and after the ten cycles. Both values are largely retained (*p*-type: +51 → +51 μV/K; *n*-type: −58 → −56 μV/K), consistent with good thermal stability of the active film, including the DODMAC-stabilized *n*-type doping.
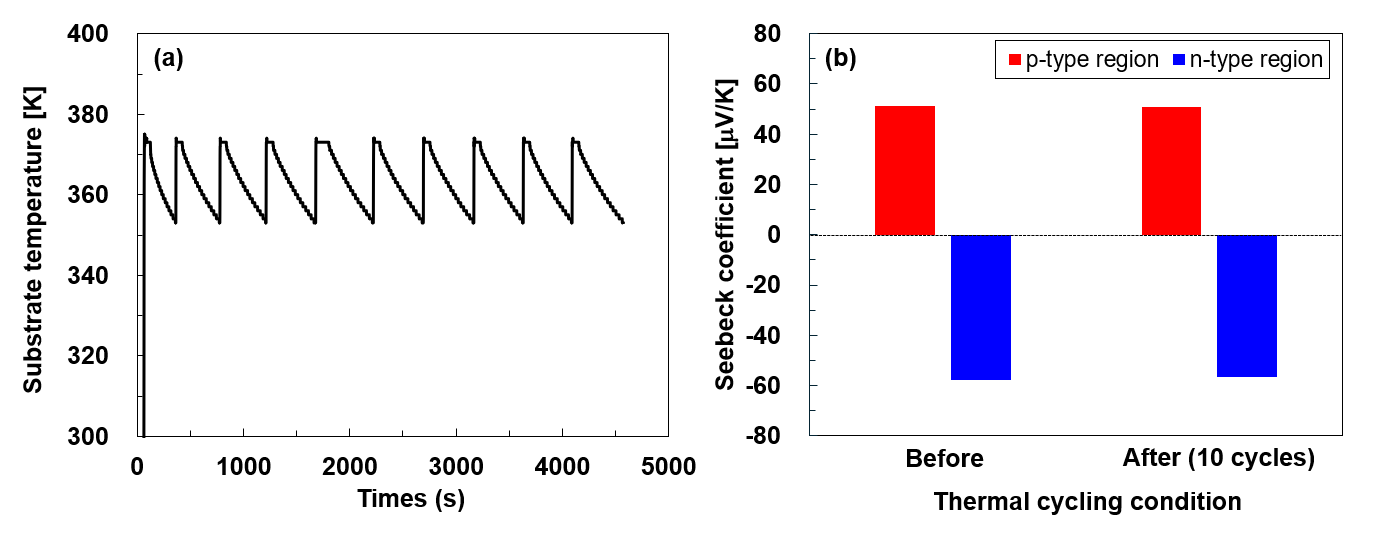


For the thermal-cycling test, the device was subjected to ten consecutive heating–cooling cycles in which the substrate temperature was raised rapidly to ≈ 375 K and then allowed to relax to ≈ 350 K [Fig. S8(a)], the entire sequence spanning ≈ 4600 s. The peak temperature was set 50 K above the 325 K device operating temperature so as to impose a stress more demanding than that encountered in normal operation. The cycling was deliberately confined to the elevated 350–375 K band, rather than returned to room temperature on each cycle, so as to keep the film continuously within the high-temperature regime where any thermally activated degradation of the DODMAC-stabilized *n*-type doping would be expected to occur, thereby probing the doping stability under a sustained, rather than intermittent, high-temperature exposure. Comparison of the Seebeck coefficients before and after cycling [Fig. S8(b)] shows that both regions are largely retained, with the *n*-type magnitude decreasing only marginally from 58 to 56 μV/K. Because the air-stable *n*-type behavior relies on DODMAC doping, whose thermal stability is the specific concern for cyclic operation, this result is consistent with the *n*-type doping—and hence the active *p–n* junction underlying both operating modes—remaining stable under repeated thermal cycling above the operating temperature.

Taken together, the bending and thermal-cycling results indicate that the spatial Seebeck profile of the active film, which governs the output voltage in both the power-generation and heat-flux-sensing modes, is largely retained under both mechanical and thermal stress. Because the operating mode is selected by the substrate—whose optical and thermal properties are intrinsic and not expected to change appreciably under these tests—the retained Seebeck profile supports the expectation that the voltage response of both the COP-based power-generation device and the PEN-based sensing device is largely maintained under cyclic operation.

**S8. Transient thermal model**

To provide quantitative support for the substrate-dependent mechanism (main text, Section 4.3 and Fig. 7) and to separate the heat-transfer pathways inherent to the hot-plate measurement, we developed a reduced-order one-dimensional transient thermal model of the in-plane temperature distribution along a single *p–n* strip. The strip is discretized along its long axis *x*, with the junction at x = 0 and the outer ends at *x* = ±*L* (*L* = 20 mm). Because the SWCNT film exhibits a far higher in-plane thermal conductivity than the substrates (Table 1, main text), the film is treated as a thin sheet whose local temperature *T*(*x*,*t*) obeys

$C\frac{\partial T}{\partial t} = k_{f}d_{f}\frac{\partial^{2}T}{\partial x^{2}} + U_{b} [T_{\mathrm{drive}}(t) - T] + G(t) \cos(\pi x/L)$ (S1)

where *C* = *ρ*_f_ *c*_f_ *d*_f_ is the areal heat capacity of the film and *k_f_* *d*_f_ its in-plane sheet conductance. The measured device temperature of each substrate (Fig. 4(a), main text) is supplied directly as the driving term *T*_drive_ (*t*) through the coupling coefficient *U*_b_. Because the devices were heated in full contact with the hot plate, heat enters the back face essentially uniformly; this common term therefore aggregates the heat-transfer contributions shared by all three devices—contact conduction, convective exchange with the ambient, and the hot-plate overshoot, which is contained in the measured *T*_drive_(*t*). The substrate-specific in-plane gradient is represented by the zero-mean source term *G*(*t*) cos(π*x*/*L*), with

$G(t) = g_{c} \tau(\lambda) \varphi(t) - g_{e} \alpha(\lambda) \Pi^{p} \psi(t)$ (S2)

Here *τ*(λ) and *α*(λ) are the substrate transmittance and absorptance at λ = 8.8 μm (Table 1, main text; original FT-IR spectra in Fig. S2), and *Π* is the per-unit-area thermal inertia relative to PI. The first term represents the COP regime: infrared radiation transmitted through the weakly absorbing COP substrate (*τ* = 45.8%) heats the SWCNT film preferentially, and, because this preferential central heating persists under steady illumination, a steady center-hot gradient is sustained, described by the dimensionless envelope *φ*(*t*) (normalized to unity at steady state). The second term represents the PI/PEN regime: because these substrates absorb almost all the incident infrared (*α* ≈ 1, as confirmed by the measured absorbance of 2.7–3.0 at 8.8 μm, corresponding to a transmittance below 0.2%; Fig. S2), the exposed substrate margins surrounding the film are heated directly and conduct laterally into the film ends, transiently inverting the gradient. The dimensionless transient envelope *ψ*(*t*) is the excess of *T*_drive_(*t*) above its slowly varying trend, normalized to its peak value (a measure of the instantaneous heating rate); it peaks during the rapid heating phase and decays to zero at thermal equilibrium, consistent with the near-zero steady-state voltages of PI and PEN. The exponent *p* ≈ 0.6 reflects the sub-linear (diffusive, ∝ √*Π*) growth of the inverted gradient with substrate thermal inertia.

Physically, the terms of Eqs (S1) and (S2) correspond directly to the heat-transfer pathways of the hot-plate measurement. In Eq (S1), the storage term *C* ∂*T*/∂*t* represents the rate at which heat accumulates in the film (its thermal inertia); *k*_f_ *d*_f_ ∂²*T*/∂*x*² represents in-plane heat conduction, which redistributes heat along the strip and tends to flatten the gradient; *U*_b_[*T*_drive_(*t*) − *T*] represents the substrate-mediated coupling to the measured device temperature and therefore lumps together the contributions common to all three devices—contact conduction from the hot plate, convective exchange with the surrounding air, and the hot-plate overshoot (carried by the measured *T*_drive_(*t*)); and *G*(*t*) cos(π*x*/*L*) represents the substrate-specific photothermal source—the infrared-absorption term—which is the only contribution that breaks the in-plane symmetry and therefore sets the direction of the gradient. In Eq (S2), the first term *g*_c_ *τ*(*λ*) *φ*(*t*) is the transmission (COP) channel, in which infrared passes through the weakly absorbing substrate and heats the film at its center, while the second term −*g*_e_ *α*(*λ*) *Π^p^* *ψ*(*t*) is the absorption (PI/PEN) channel, in which infrared is absorbed at the substrate margins and heats the outer ends, with an amplitude that scales with the substrate thermal inertia *Π*.

The two gain coefficients *g*_c_ and *g*_e_ quantify the effective areal photothermal power coupled into the transmission and absorption channels, respectively. Because *τ*(*λ*), *α*(*λ*), *φ*(*t*), and *ψ*(*t*) are all dimensionless, *g*_c_ and *g*_e_ carry the units of the source term in Eq (S1)—namely W/m²—and are expected to be of the order of the net infrared flux exchanged near room temperature (tens of W/m²); their calibrated values, *g*_c_ = 53.3 W/ m² and *g*_e_ = 2.87 W/m², are consistent with this expectation. The exponent *p* is dimensionless and controls how strongly the inverted-gradient amplitude grows with the relative substrate thermal inertia *Π*; it is expected to lie between 0.5 (purely diffusive growth, ∝ √*Π*) and 1 (linear growth), and the calibrated value *p* = 0.6 indicates near-diffusive behavior.

This reduced-order description rests on three approximations. First, a one-dimensional treatment is adopted: because each strip is long and narrow (40 mm × 10 mm) and the copper leads at its two ends define the only in-plane heat paths of interest, the temperature is taken to vary solely along the long axis *x*, with transverse variation neglected. Second, a thin-film (lumped through-thickness) approximation is used: the SWCNT film is sufficiently thin that its through-thickness thermal-diffusion time (well below one second) is far shorter than the minute-scale voltage transients of Fig. 4(b), so the film is essentially isothermal across its thickness and can be represented as a single sheet of areal heat capacity *C* and sheet conductance *k*_f_ *d*_f_. Third, the outer ends are treated as adiabatic (∂*T*/∂*x* = 0), so that the direction and shape of the in-plane gradient are set by the spatial distribution of the photothermal source [the *G*(*t*) cos(π*x*/*L*) term] rather than by end heat-sinking; this is a conservative choice, since the modest additional heat-sinking provided by the copper leads in the real device would only reinforce the same center-hot (COP) or center-cold (PI/PEN) gradient, leaving the decoupling conclusions of Fig. 7 unchanged.

Equation (S1) is solved by the method of lines on a uniform grid of 81 nodes using an implicit (BDF) time integrator. The open-circuit voltage of the series-connected device is obtained from the measured Seebeck profile *S*(*x*) (Fig. 3(a), main text) as

$V(t) = 2 \int_{-L}^{+L} S(x) (\frac{\partial T}{\partial x}) dx$ (S3)

The model contains only three adjustable coefficients (*g*_c_, *g*_e_, *p*); all remaining quantities are taken from the measured material properties (Table 1, main text) and from the measured temperature and Seebeck profiles. The three coefficients were fixed once, by matching three measured voltages of Fig. 4(b): the COP steady-state voltage (+0.38 mV, the *t* = 12–15 min steady-state plateau, sustained through *t* = 30 min in the extended single-device run) and the PI and PEN transient peak voltages (−0.58 and −0.96 mV, both occurring at *t* ≈ 6 min, approximately one minute after heater activation at *t* ≈ 5 min). With these values fixed, the full temporal evolution of the output voltage, the near-zero steady-state voltages of PI and PEN, the spatial temperature profiles (Fig. S10), and the decoupling behavior (main text, Fig. 7) are reproduced without further adjustment. The model parameters are shown in Table S2.

Table S1. Optical and thermal properties of the Kapton double-sided adhesive tape (No. 760H #25, Teraoka Seisakusho Co.).

| **Property** | **Value** | **Source / method** |
| --- | --- | --- |
| Thickness | 145 μm | Manufacturer specification |
| Absorbance at 8.8 μm | 2.96 | FT-IR (this work) |
| Transmittance at 8.8 μm | 0.11 % | FT-IR (this work) |
| Thermal conductivity, *κ* | ≈0.15 W/(m·K) | Literature estimate (composite)* |
| Specific heat, *c*_p_ | ≈1.3 J/(g·K) | Literature estimate (composite)* |

* Estimated as a layered composite of the 25-μm Kapton (polyimide) base film (*κ* = 0.12 W/(m·K), *c*_p_ = 1.09 J/(g·K); DuPont Kapton HN datasheet) and the ~120-μm silicone adhesive layers (*κ* = 0.15–0.20 W/(m·K), *c*_p_ = 1.2–1.5 J/(g·K); representative literature values for silicone elastomers), using a series model for the through-thickness conductivity and a mass-weighted average for the specific heat. The manufacturer's datasheet does not provide thermal properties.

Because the same adhesive tape—identical in material, thickness, and bonding procedure—is used in all three devices (COP, PI, and PEN), its contributions to infrared absorption, interfacial thermal resistance, and thermal inertia are common to the three devices. In the transient thermal model (Section S8), these common contributions are carried by the measured driving temperature *T*_drive_(*t*) and therefore enter the *U*_b_[*T*_drive_(*t*) − *T*] term rather than the substrate-specific source *G*(*t*) cos(π*x*/*L*). Consequently, the adhesive layer cannot account for the substrate-dependent polarity reversal: in the matched-optics numerical experiment (main text, Fig. 7(a)), in which only the substrate optical properties are equalized while this common term is left unchanged, the polarity reversal disappears entirely. The adhesive nevertheless represents a parasitic infrared-absorption pathway, and replacing it with an infrared-transparent bonding layer is identified in the main text (Section 4.4) as a route to strengthen the photothermal driving force.

Table S2. Parameters of the transient thermal model.

| **Symbol** | **Description** | **Value / source** |
| --- | --- | --- |
| *L* | Half-length of the strip (junction to outer end) | 20 mm |
| *k*_f_ *d*_f_ | Film in-plane sheet conductance | *k*_f_ = 3.4 W/( m·K) (Table 1); *d*_f_ = 45 μm |
| *C* = *ρ_f_* c*_f_* d*_f_* | Film areal heat capacity | *ρ_f_* *c_f_* ≈ 4 × 10⁵ J/(m³·K); *d*_f_ = 45 μm |
| *U*_b_ | Coupling to the measured device temperature | 8 W/(m²·K) |
| *τ*(*λ*), *α*(*λ*) | Substrate transmittance / absorptance at 8.8 μm | Table 1; measured spectra in Fig. S2 (COP *τ* = 45.8%; PI/PEN *α* ≈ 1) |
| *Π* | Per-unit-area thermal inertia (relative to PI) | PI = 1.0; PEN = 2.4 (Table 1) |
| *g*_c_, *g*_e_, *p* | Calibrated coefficients | *g*_c_ = 53.3 W/m²; *g*_e_ = 2.87 W/m²; *p* = 0.6 (dimensionless); fixed to the COP/PI/PEN voltages |

The film in-plane thermal conductivity (*k_f_* = 3.4 W/(m·K)) and thickness (*d_f_* = 45 μm), as well as the areal heat capacity, were taken as the mean of the *p*-type and *n*-type SWCNT film values listed in Table 1 of the main text, since a single composite strip comprises both regions in series.

**
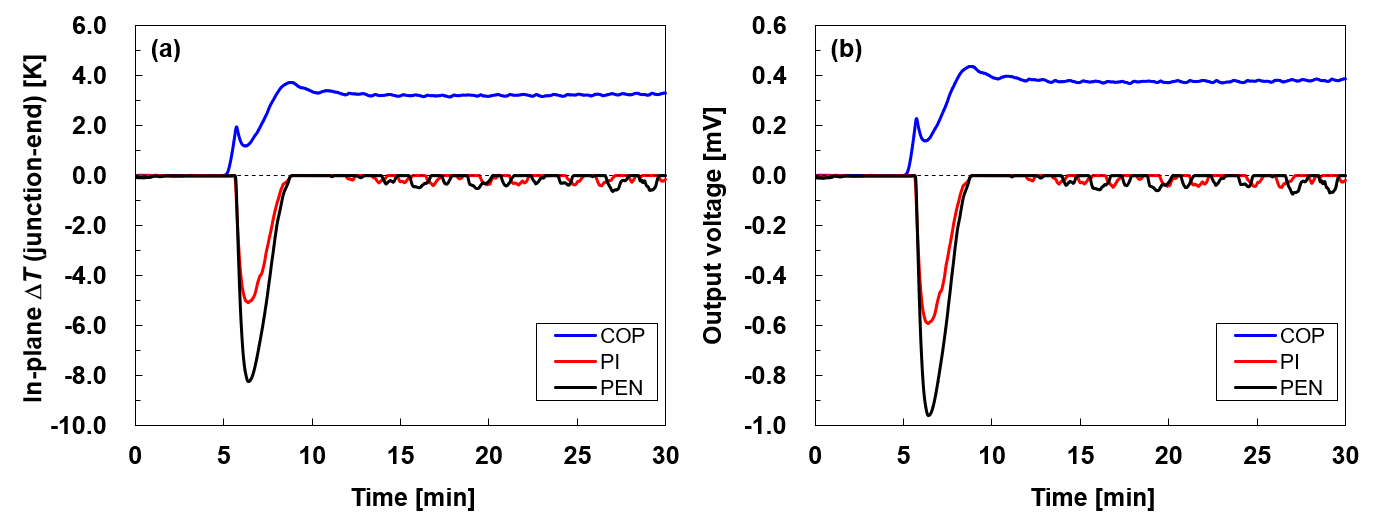
Figure S9.** Comparison between the thermal model and experiment. (a) Computed in-plane temperature difference Δ*T* = *T*_junction_ − *T*_outer_end_ along the *p–n* strip for the COP, PI, and PEN devices. (b) Corresponding output voltage obtained from the measured Seebeck profile (Fig. 3(a), main text); open circles denote the measured steady-state voltages (Table 2, main text). The model reproduces the sustained positive response of COP and the transient negative excursions of PI and PEN, including their relaxation toward zero at steady state.

**
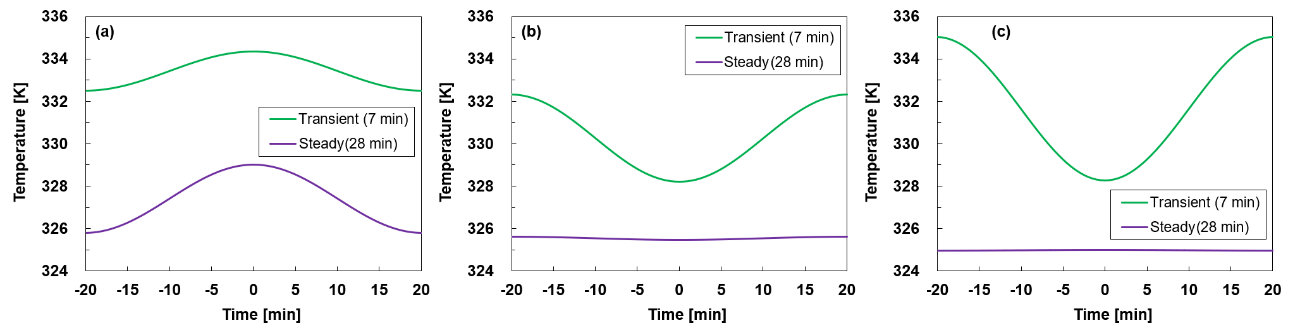
**

**Figure S10.** Computed in-plane temperature profiles *T*(*x*) along the strip (junction at *x* = 0) during the transient (7 min) and at steady state (28 min). COP maintains a bell-shaped, center-hot profile (power-generation gradient), whereas PI and PEN develop a transient valley-shaped, center-cold profile (inverted, heat-flux-sensing gradient) that is deepest for PEN owing to its larger thermal inertia; both relax toward uniformity at steady state. These profiles correspond to the thermographic maps of Fig. 5 and the schematic of Fig. 6 in the main text.
